# Extraction of the Anticancer and Antimicrobial Agent, Prodigiosin, from *Vibrio gazogenes* PB1 and Its Identification by 1D and 2D NMR

**DOI:** 10.3390/molecules27186030

**Published:** 2022-09-16

**Authors:** Dhanya Vijay, Nassra S. Alshamsi, Ziad Moussa, M. Kalim Akhtar

**Affiliations:** Department of Chemistry, College of Science, United Arab Emirates University, Al Ain P.O. Box 15551, United Arab Emirates

**Keywords:** secondary metabolite, prodiginine, marine bacteria, biotechnology, anticancer, ^1^H, ^13^C

## Abstract

Prodigiosin is a secondary metabolite produced in several species of bacteria. It exhibits antimicrobial and anticancer properties. Methods for the extraction and identification of prodigiosin and their related derivatives from bacterial cultures typically depend on solvent-based extractions followed by NMR spectroscopy. The estuarine bacterium, *V. gazogenes* PB1, was previously shown to produce prodigiosin. This conclusion, however, was based on analytical data obtained from ultraviolet-visible absorption spectrophotometry and infrared spectroscopy. Complete dependence on these techniques would be considered inadequate for the accurate identification of the various members of the prodiginine family of compounds, which possess very similar chemical structures and near-identical optical properties. In this study, we extracted prodigiosin from a culture of *Vibrio gazogenes* PB1 cultivated in minimal media, and for the first time, confirmed the synthesis of prodigiosin *Vibrio gazogenes* PB1 using NMR techniques. The chemical structure was validated by ^1^H and ^13^C NMR spectroscopy, and further corroborated by 2D NMR, which included ^1^H-^1^H-gDQFCOSY, ^1^H-^13^C-gHSQC, and ^1^H-^13^C-gHMBC, as well as ^1^H-^1^H-homonuclear decoupling experiments. Based on this data, previous NMR spectral assignments of prodigiosin are reaffirmed and in some cases, corrected. The findings will be particularly relevant for experimental work relating to the use of *V. gazogenes* PB1 as a host for the synthesis of prodigiosin.

## 1. Introduction

**Figure 1:**
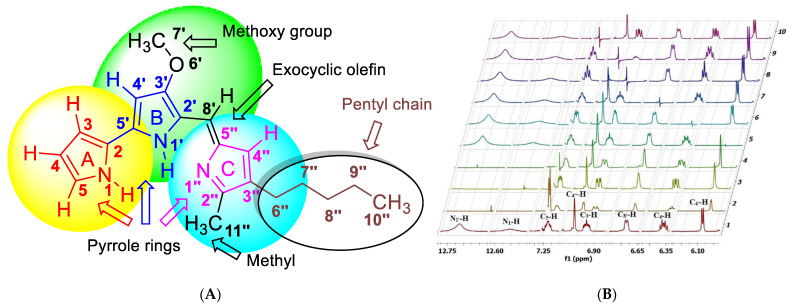
NMR analysis of prodigiosin. (**A**) Chemical structure of prodigiosin indicating the numbers and positions of the individual atoms. (**B**) ^1^H-^1^H-Homonuclear decoupling experiments of prodigiosin. (1) Non-decoupled spectrum; (2) multifrequency decoupling of N_1_-H and N_1’_-H; (3) irradiation of N_1_-H; (4) irradiation of N_1’_-H; (5) irradiation of C_4’_-H; (6) irradiation of C_4_-H; (7) irradiation of C_8’_-H; (8) irradiation of C_3_-H; (9) irradiation of C_4’’_-H; (10) irradiation of C_5_-H.

Prodigiosin is a secondary metabolite that belongs to the prodiginine family and has received renewed attention due to its diverse biotechnological applications [1]. It has potential uses as an anticancer agent [2], antibiotic [3], and fungicide [4]. The structure of prodigiosin was first ascertained from total synthesis and later from product isolation [5]. The distinctive red colour of prodigiosin is attributed to the highly conjugated system of its three pyrrole rings (A–C). Ring B bears a methoxy substituent at the C_3′_ position, while ring C is attached to an exocyclic olefin at C_5’’_ and is substituted at the C_2’’_ and C_3’’_ by a methyl group and a hydrophobic pentyl chain, respectively (Figure 1A).

Prodigiosin is produced in several species of bacteria, including *Serratia marcescens* and *Hahella chejuensis*, and *Pseudoalteromonas rubra* [6,7,8]. By far, the most commonly employed bacterium for the production of prodigiosin is *S. marcescens* [1,3,5,9,10,11,12,13]. We recently demonstrated that *Vibrio gazogenes* PB1, previously known as *Beneckea gazogenes* [14], possesses excellent physiological traits as a bacterial host for the production of prodigiosin in minimal media [15]. As a host for prodigiosin production, *V. gazogenes* PB1 is appealing for a number of reasons. Firstly, this particular bacterial species is widely available from several culture collections around the world, including the Korean Collection for Type Cultures (KCTC: 12695). Secondly, *V. gazogenes* can tolerate and grow in saline conditions. This is a useful trait from an industrial perspective, as it would hinder the growth of microbial contaminants during a scale-up [16]. Thirdly, *V. gazogenes* is a biosafety level 1 organism, and would therefore pose a low risk for large-scale use and for research purposes. In contrast, the commonly used *S. marcescens* is an opportunistic pathogen known to causes infections in humans [17].

At the time of its discovery, only ultraviolet-visible absorption spectrophotometry and infrared spectroscopy were used to confirm the synthesis of prodigiosin in *V. gazogenes* PB1 [14]. Though such techniques are relatively quick, simple, non-destructive, and require only a small amount of sample, complete dependence on these techniques would be insufficient for the accurate identification of prodiginine compounds, which possess very similar chemical structures and near-identical optical properties. For example, the maximum absorption wavelength of prodigiosin and undecylprodigiosin compounds are 535 nm and 533 nm, respectively [18,19]. Furthermore, both of these prodiginine analogs possess exactly the same number and type of functional groups and would be difficult to distinguish using basic analytical techniques. 

For the structural validation of secondary metabolites such as prodigiosin, one-dimensional nuclear magnetic resonance (1D NMR) has been a long-favored method [18]. The overlap of signals encountered in 1D NMR, however, can sometimes confound the validation of structures for even small complex molecules; this can be overcome with the use of two-dimensional NMR (2D NMR) techniques such as double-quantum filtered correlation spectroscopy (gDQFCOSY), heteronuclear single-quantum correlation spectroscopy (gHSQC) and heteronuclear multiple-bond correlation (HMBCAD), which offer a superior level of structural details [20]. Herein, we confirm for the first time the synthesis of prodigiosin isolated from a culture of *V. gazogenes* PB1 using both 1D and 2D NMR techniques. Additionally, we provide evidence based on 1D and 2D NMR analyses to correct current chemical assignments for prodigiosin, as well as reassign inaccurate ones.

## 2. Results

### 2.1. Extraction of Prodigiosin from Bacterial Cultures

For the extraction of prodigiosin, the following commonly used laboratory organic solvents were employed: acetone, methanol, ethanol, chloroform, and dichloromethane. In agreement with an earlier study [21], acetone was indeed found to be the most superior solvent for the extraction of prodigiosin, with both ethanol and methanol offering very similar extraction performances (Figure S1A, Supplementary Materials). With respect to drying times, acetone offered the quickest drying time of around 2 h, while ethanol and methanol required 4 to 5 h of drying time (Figure S1B, Supplementary Materials). The amount of prodigiosin extracted was determined by recording the absorbance at 535 nm [18] and comparing this to the amount of prodigiosin extracted using acetone, which had previously been shown to result in the best extraction (Figure S2, Supplementary Materials) [21]. Having ascertained that acetone was the most superior extraction solvent, this solvent was therefore used to prepare the prodigiosin extract for NMR analysis.

### 2.2. 1D NMR Analysis of Prodigiosin

An examination of the ^13^C-CRisis-Attached Proton Test (^13^C-CRAPT) NMR confirmed the presence of the expected 20 carbon signals: five aromatic CHs, one olefinic CH, seven aromatic sp^2^ hybridized quaternary carbons, one methoxy, two methyls, and four methylene carbons, which is consistent with all carbon atoms being magnetically nonequivalent (Table 1, Figures S3 and S4). The ^13^C-CRAPT spectrum showed seven signals in the aliphatic region, thus, confirming the presence of the four pentyl chain methylenes as indicated by the positive phases of the corresponding signals for C_6’’_-C_9’’_ (δ 31.4, 29.8, 25.3, 22.5 ppm). In the same region, three signals with negative phases supported the presence of a methoxy group (C_7’_: δ 58.7 ppm) and two methyl carbons C_10’’_ and C_11’’_ (δ 14.3 & 12.5 ppm). The methoxy group was readily assigned given its distinctive downfield chemical shift at δ 58.7 ppm. The integration of the ^1^HNMR spectrum confirmed the presence of 25 protons: six aromatic and olefinic Hs, two NHs, and 17 aliphatic protons (Table 1, Figure S5, Supplementary Materials). The peaks obtained for the ^13^C NMR analyses (101 MHz, deuterated chloroform (CDCl_3_) and ^1^H NMR (400 MHz, CDCl_3_) are summarized in Table 1.

### 2.3. 2D NMR Analysis of Prodigiosin

To fully assign the ^1^H NMR resonances of the two methyl groups on the aliphatic pentyl chain (C_10’’_) and pyrrole C ring (C_11’’_) and correlate their chemical shifts to the respective attached carbons, we resorted to 2D NMR by employing several techniques. Based on the ^1^H-^13^C-gHSQC 2D spectrum, the C_10’’_H_3_ methyl (*δ* 0.89), which appears as a triplet in the ^1^H-NMR, was correlated to the carbon at *δ* 14.3 ppm (off-diagonal cross peak at *δ* 0.89/14.3 ppm), while the C_11’’_H_3_ methyl singlet in the ^1^H-NMR matched the carbon at *δ* 12.5 ppm (off-diagonal cross peak at *δ* 2.54/12.5 ppm) (Figure S6, Supplementary Materials). The pentyl methylenes were identified in the ^1^H-NMR based on their multiplicity pattern and were correlated using ^1^H-^1^H-gDQFCOSY. They were matched with the corresponding carbon signals using ^1^H-^13^C-gHSQC. The distinctive and only expected methylene triplet of C_6’’_H_2_ at *δ* 2.39 ppm (t, 2H, *J* = 7.6 Hz) triggered the assignment of the remaining CH_2′_s (C_7’’_H_2_- C_9’’_H_2_) using ^1^H-^1^H-gDQFCOSY (Figure S7A–D, Supplementary Materials). Thus, a strong contour between C_6’’_H_2_ and the adjacent C_7’’_H_2_ (*δ* 2.39/1.53 ppm) confirmed their vicinal relationship and is further supported by the anticipated quintet appearance of C_7’’_H_2_ (quint, 2H, *J* = 7.6 Hz) and matching scalar couplings. The latter is further correlated to C_8’’_H_2_ and C_9’’_H_2_, which appear as a broad complex multiplet with a chemical shift range between *δ* 1.37–1.21 ppm. This multiplet clearly correlates to the terminal pentyl methyl C_10’’_H_3_ with a strong cross peak (*δ* 1.37–1.21/0.89 ppm). Further, ^1^H-^1^H-gDQFCOSY correlated the right-hand side of the complex signal (*δ* 1.53/1.29–1.21 ppm) with C_7’’_H_2_, confirming the chemical shift of C_8’’_H_2_ as the latter, which was also correlated through ^1^H-^13^C-gHSQC to C_8’’_ (*δ* 1.29–1.21/31.4 ppm). On the other hand, the left-hand side portion of the broad signal at *δ* 1.37–1.21 ppm (*δ* 1.37–1.29 ppm) was assigned to C_9’’_H_2_ and correlated, via ^1^H-^13^C-gHSQC, to C_9’’_ (*δ* 1.37–1.29/22.5 ppm). With these assignments in hand, matching the methylene protons C_6’’_H_2_-C_9’’_H_2_ to the corresponding carbon atoms using ^1^H-^13^C-gHSQC and ^1^H-^1^H-gDQFCOSY was straightforward (C_6’’_H_2_: *δ* 25.3; C_7’’_H_2_: *δ* 29.8; C_8’’_H_2_: *δ* 31.4; C_9’’_H_2_: *δ* 22.5) (Figures S6 and S7A–D). 

The aromatic CHs of ring A (C_3_H-C_5_H) were clearly distinguished as one spin system in the ^1^H-^1^H-gDQFCOSY spectrum, which shows two contours correlating C_4_H with both, C_3_H (*δ* 6.92/6.36 ppm) and C_5_H (*δ* 7.23/6.36 ppm) (Figure S7A–D). Although ^1^H-^1^H-gDQFCOSY correlations confirmed the chemical shift of C_4_H, distinguishing between C_3_H and C_5_H was more challenging. In principle, ^1^H-^1^H-ROESY correlation between N_1_-H and C_5_H could identify the chemical shift of the latter. Unfortunately, ^1^H-^1^H-ROESY (Figure S8, Supplementary Materials) displayed very weak signals due to low sample concentration and could not be used for data analysis. The suggested assignment of C_3_H and C_5_H is supported by considering the negative inductive effect (-I) of N_1_ as well as its negative mesomeric effect. Based on the latter phenomenon, C_5_ (directly bonded to the more electronegative N atom compared to C_3_, which is connected to the C atom) is expected to resonate much further downfield in the ^13^C than C_3_. Therefore, the ^13^C signals at δ 117.0 and 127.0 ppm, which were correlated through the ^1^H-^13^C-HSQC to the unassigned proton chemical shifts at δ 6.92 and 7.23 ppm, respectively, suggest that the cross-peak at δ 6.92/117 ppm stems from C_3_H and the contour at (δ 7.23/127 ppm) belongs to the more shifted C_5_H. Further strong evidence supporting this assignment is based on the negative mesomeric effect of N_1_, which delocalizes electrons to C_3_, causing a significant ^13^C upfield chemical shift of C_3_ (δ 117.0 ppm). Notably, the C_3_H is also shifted upfield relative to C_5_H. Such high upfield shifts are well documented for carbons and protons in the ortho position of aromatic alcohols, alkoxy, and amine compounds where ^13^C chemical shifts resonate typically around δ 116 ppm.

The unexpected observation of two strong contours in the ^1^H-^1^H-gDQFCOSY between C_3_H (*δ* 6.92)/C_5_H (*δ* 7.23) and the chemical shift of a broad signal at *δ* 12.56 ppm confirmed the latter absorption as the N_1_-H of ring A. Most intriguing was the contribution of N_1’_-H (*δ* 12.71 ppm) of the pyrrole ring B, which led to the most challenging task of assigning the chemical shifts of C_4’_H, C_8’_H, and C_4’’_H. 

The presence of two strong cross-peaks due to long-range coupling (^4^*J*_N’H-CH_) in the ^1^H-^1^H-gDQFCOSY between N_1’_-H and the two doublets at *δ* 6.68 (*J* = 2.4 Hz) and 6.08 (*J* = 2.0 Hz) ppm immediately established that these chemical shifts are related to C_4’_H and C_8’_H. Hence, the only remaining and unassigned signal at *δ* 6.96 must belong to the C_4’’_H singlet, which is clearly an isolated uncoupled spin system. Interestingly, the C_4’’_H showed ^1^H-^13^C heteronuclear multiple-bond correlation (^3^*J*_H-C-13_) in the ^1^H-^13^C-HMBCAD spectrum with C_8’_ (*δ* 128.4 ppm), which in turn was traced to *δ* 6.68 using gHSQC and assigned to C_8’_H, indicating that C_4’_H resonates at *δ* 6.08 (^13^C: *δ* 92.8 ppm) (Figure S9A–E, Supplementary Materials). The methoxy group is well known to cause an extreme upfield shift of the ^13^C and ^1^H chemical shifts of *ortho* carbons and protons, as observed with C_4’_H, thereby lending further support to the suggested assignment. The proton chemical shifts in the skeletal structure of prodigiosin were subsequently traced to the attached carbons using gHSQC, as summarized in Table 1.

The quaternary carbons of ring C, C_2’’_, C_3’’_, and C_5’’_ were assigned δ 128.5, 147.1, and 125.2 ppm, respectively, based on the strong ^1^H-^13^C-HMBC correlation of C_11’’_-H_3_ with C_3’’_ (^3^*J*_CH_: δ 12.5/147.1 ppm), C_6’’_-H_2_/ C_4’’_-H with C_2’’_ (^3^*J*_CH_: δ 6.96 & 2.39/128.5 ppm), and C_8’_-H with C_5’’_ (^2^*J*_CH_: δ 6.68/125.2 ppm). On the other hand, the quaternary carbons of ring B, C_2’_, C_3’_, and C_5’_ and were assigned δ 120.7, 165.7, and 147.7 ppm, respectively, based on strong ^1^H-^13^C-HMBC correlation of C_4’_-H with C_2’_ (^3^*J*_CH_: δ 6.08/120.7 ppm) and C_5’_ (^2^*J*_CH_: δ 6.08/147.7 ppm), and C_7’_-H_3_ with C_3’_ (^3^*J*_CH_: δ 4.01/165.7 ppm). Notably, the ^3^*J*_CH_ correlation contour between C_4’_-H and C_2’_ (^3^*J*_CH_: δ 6.08/120.7 ppm) was much stronger compared to the ^2^*J*_CH_ cross-peak observed between C_4’_-H and C_5’_ (^2^*J*_CH_: δ 6.08/147.7 ppm), which is typical in HMBC spectra. Further evidence to support such an assignment is based on the phenomenon of negative mesomeric effect of the methoxy substituent, which severely shifts carbon and proton signals upfield. Such high upfield shifts are well documented for carbons and protons in the ortho position of aromatic alkoxides where ^13^C chemical shifts typically appear around *δ* 116 ppm. In this case, C_2′_ resonates at 120.7 ppm. Furthermore, the same double mesomeric effect of the methoxy substituent and nitrogen atom on C_4′_ caused even a more severe upfield shift of C_4′_ to 92.8 ppm. Another convincing factor is that C_5′_ is directly connected to a more electronegative atom (N_1′_-C_5′_), causing extreme downfield shift of the C_5′_ to 147.7 ppm. Finally, the remaining unassigned chemical shift at δ 122.3 was attributed to C_2_ of Ring A.

### 2.4. ^1^H-^1^H-Homonuclear Decoupling Experiments

The ^1^H-^1^H-gDQFCOSY cross-peaks were insufficient to totally correlate the C_3_-H-C_5_-H spin system and unambiguously match the ^1^HNMR chemical shifts of ring A to the C_3_-H-C_5_-H protons because of the weak correlation of C_5_-H with C_4_-H in the gDQFCOSY, and the close overlap between the ddd signals for each of the ring A protons. As some of the key coupling constants required for unequivocal assignments were still unresolved, homonuclear decoupling experiments were carried out to match the chemical shifts of ring A protons with their respective positions on the aromatic ring, calculate the J-values, and provide further evidence for the remaining assignments (Figure 1B and Figure S10A–K).

The original, full spectrum is shown in Figure 1B as spectrum 1. Starting with ring A protons, irradiation of C_5_-H (Figure 1B spectrum 10), C_4_-H (Figure 1B, spectrum 6), C_3_-H (Figure 1B, spectrum 8), and N_1_-H (Figure 1B, spectrum 3) resulted in C_4_-H and C_3_-H collapsing into a dd (Figure 1B, spectrum 10), C_3_-H and C_5_-H collapsing into a dd (Figure 1B, spectrum 6), C_4_-H and C_5_-H collapsing into a dd (Figure 1B, spectrum 8), and C_3_-H, C_4_-H, and C_5_-H collapsing into a dd (Figure 1B, spectrum 3), respectively. Even more intriguing was the collapse of C_4’_-H and C_8’_-H into singlets (Figure 1B, spectrum 4) when N_1’_-H was decoupled, proving their proximity and they comprise an independent long-range coupled spin system. 

As expected, decoupling of C_4’_-H (Figure 1B, spectrum 5) and C_8’_-H (Figure 1B, spectrum 7) had no impact. The preceding experiments led to the unambiguous assignment of the chemical shifts for ring A and B protons through their scalar coupling constants and splitting pattern. Based on spectrum 10, the long-range coupling constant ^4^*J (*N_1_-H-C_3_-H) and ^4^*J (*N_1_-H-C_4_-H) is 2.4 Hz and ^3^*J (*C_3_-H-C_4_-H) is 4.0 Hz. From spectrum 3, ^3^*J* (C_3_-H-C_4_-H) is 4.0 Hz, ^3^*J* (C_4_-H-C_5_-H) is 2.4 Hz, and ^4^*J* (C_3_-H-C_5_-H) is 1.6 Hz. According to spectrum 6, ^3^*J (*N_1_-H-C_5_-H) is 1.2 Hz. Thus, the total *J*-coupling values are *δ* 6.92 (ddd, *J* = 4.0, 2.4, 1.2 Hz), 6.36 (ddd, *J* = 4.0, 2.4, 2.4 Hz), and 7.23 (ddd, *J* = 2.4, 1.6, 1.2 Hz). The J values support the earlier assignment of ring A-C proton positions and confirm the presence of ^3^*J* and long-range couplings of N_1_-H and N_1’_-H, which were key to full and successful structural elucidation. As expected, the decoupling of the C_4’’_-H singlet (Figure 1B, spectrum 9) had no impact on the spectrum compared to the uncoupled one (Figure 1B, spectrum 1).

## 3. Discussion

In this study, we show that an acetone-based extraction method for prodigiosin from a culture of *V. gazogenes* PB1 was adequate for downstream NMR analysis, which is often a critical step in validating the structure of secondary metabolites [22]. NMR was employed for the following reasons: (i) it is well suited for target chemicals that are not commercially available or costly to source [23], (ii) it does not require derivatization, nor extensive sample separation, [23], and (iii) it does not depend on an elaborate instrument set-up [23].

The miscible solvents (acetone, ethanol and methanol) were found to be the most effective solvents for prodigiosin extraction in accordance with an earlier study [21]. Of the three, acetone is ideal given its faster drying time. Surprisingly, the sole use of the immiscible solvents, chloroform, and dichloromethane, resulted in the poorest extraction even though these solvents possess a hydrophobic nature like that of prodigiosin. The ‘like-for-like’ approach for extraction clearly does not hold well in this case. This seemingly peculiar result is most likely attributed to the physiology of bacterial membranes. Bacteria naturally secrete a hydrophilic layer of polysaccharides over their membrane surface, known as the capsule [24]. This can serve a number of functions, including cell-to-cell attachment, cell-to-cell communication and even evading the immune response of its host. Immiscible solvents such as chloroform will be unable to effectively strip away this hydrophilic layer due to the lack of hydrogen bonding. Much of the prodigiosin will most probably remain trapped within the hydrophobic portion of the bacterial membrane. Miscible solvents such as acetone will likely be more effective in dismantling the capsule due to their excellent capacity to form hydrogen bonds, whilst also solubilizing prodigiosin, via hydrophobic interaction [25].

Several publications have reported on the isolation of prodigiosin and have summarized the proton and carbon NMR data [26,27,28,29]. However, none have provided clear 2D NMR evidence to assign and match all chemical shifts to the respective H and C nuclei. While some have only reported ^1^H NMR data [27], others have provided fully assigned NMR data either without the use of 2D NMR techniques [29,30], or used insufficient 2D NMR data (COSY and HMQC only) [26]. In certain cases [26,30], a numbered structure was not provided in a figure to cross-reference the experimental ^1^H and ^13^C data provided by the authors with the respective H and C nuclei of prodigiosin. In all cases, 2D NMR data was not made available as supporting information. The lack of adequate evidence from the available NMR analyses performed in earlier studies led to the trivial assignment of certain ^1^H and ^13^C chemical shifts. For example, the methylenes of the pentyl chain, methines of ring A, or some quaternary carbons were misassigned in earlier studies. To remove any uncertainty, herein we provide the full 2D NMR data (see supporting information section) and make convincing and complete H and C assignments of prodigiosin.

Here, we provide for the first time NMR data, based on 1D (^1^H and ^13^C CRAPT NMR), 2D NMR (^1^H-^1^H-gDQFCOSY, ^1^H-^13^C-gHSQC, and ^1^H-^13^C-gHMBC, and ^1^H-^1^H-homonuclear decoupling experiments, for the structural validation of prodigiosin isolated from a culture of *V. gazogenes* PB1. Our data is in agreement with several publications that have reported on the NMR characterization of prodigiosin in different deuterated solvents; inaccurate and erroneous assignments for prodigiosin were corrected [18,26,27,28,29]. Moreover, in contrast to previous work [26,27,28], structural assignments in this study were corroborated by 2D NMR and homonuclear decoupling experiments.

## 4. Materials and Methods

### 4.1. Extraction of Prodigiosin from V. gazogenes PB1

*V. gazogenes* PB1, obtained from the Korean Collection for Type Cultures (KCTC: 12695), was used as the bacterial source for the prodigiosin metabolite. The bacterium can also be sourced from other culture collections, as described previously by Vijay et al. [15]. For prodigiosin production, the bacterium was cultured in 20 mL minimal media supplemented with 2% (*v*/*v*) glucose and 3% (*w*/*v*) NaCl over two days in 100 mL conical flasks.

To evaluate the drying times of different organic solvents or combinations thereof for prodigiosin extraction, a 1 mL volume of bacterial culture was microfuged at 5000 *g* for 1 min at room temperature. After the removal of the supernatant, a 0.5 mL volume of the organic solvent was added to the pelleted cell. Cells were resuspended in the solvent by repeated pipetting, and vortexed vigorously for 20 s. All samples were microfuged at 15,000*g* for 5 min to spin down the cell debris. The red-coloured prodigiosin pigment present within the supernatant was quantified. A 400 µL aliquot of the supernatant was transferred to a 1.5 mL microtube and the samples were air-dried at 40 °C to determine which solvents offered the fastest drying times.

For NMR analysis, the prodigiosin was extracted from *V. gazogenes* PB1 by resuspending *V. gazogenes* PB1 cells, pelleted from a 20 mL bacterial culture in 10 mL acetone and vortexing vigorously for 30 s. After centrifugation (15,000*g* for 5 min), the supernatant, containing the prodigiosin compound, was transferred to a 10 cm glass dish. Acetone was evaporated off overnight (~18 h) at room temperature (21 °C).

### 4.2. Quantification of Prodigiosin

The levels of extracted prodigiosin were determined by absorption spectrophotometry. A 20 µL sample containing the extracted prodigiosin was added to acidified ethanol (180 µL) (1 M HCl dissolved in absolute ethanol) in a microplate well, in accordance with a previous study [18]. Absorbance was recorded at 535 nm. The amount of prodigiosin extracted for each solvent was expressed as a percentage, relative to the amount of prodigiosin extracted using acetone. This was calculated as follows: (absorbance values obtained for each solvent/absorbance values obtained with acetone) × 100. The absolute amount of prodigiosin extracted from *V. gazogenes* PB1 cell cultures was determined using the extinction coefficient of 139,800 M^−1^cm^−1^, as established in an earlier study [18]. Based on this extinction coefficient, a 0.5 to 2 mg sample was typically used for NMR analysis.

### 4.3. ^1^H and ^13^C NMR Characterisation of Prodigiosin

The dried prodigiosin extract (0.5–2 mg) was dissolved in 0.6 mL of chloroform-d (CDCl_3_), transferred to a 5 mm (OD) NMR tube and analyzed by ^1^H and ^13^C NMR at 298 K for structure verification. ^1^H and ^13^C NMR spectra were recorded on a Varian 400 spectrometer (9.4 T), observing ^1^H and ^13^C at 400 and 101 MHz, respectively, using tetramethylsilane (TMS) as the internal standard. The NMR chemical shifts (δ) are reported in parts per million (ppm) relative to the residual solvent peak (^1^H-NMR δ 7.26 for CDCl_3_; ^13^C-NMR δ 77.0 for CDCl_3_). The following abbreviations were used to explain NMR peak multiplicities: br s = broad signal, s = singlet, d = doublet, t = triplet, q = quartet, p = pentet, sept = septet, app = apparent, and m = multiplet.

## 5. Conclusions

In summary, this study describes the extraction of prodigiosin from a bacterial culture of *V. gazogenes* PB1, followed by its structural validation using 1D and 2D NMR techniques. The findings will be particularly relevant for experimental work relating to the use of *V. gazogenes* PB1 as a host for the production of prodigiosin.

## Figures and Tables

**Table 1:** ^1^H- and ^13^C-NMR data for prodigiosin.

**^1^H-NMR**	**Proton Number**	**Prodigiosin Protons ppm**	**Proton Number**	**Prodigiosin Protons ppm**
	1	12.56 (s, 1H, NH)	8′	6.68 (d, 1H, *J* = 2.4 Hz, CH)
	3	6.92 (ddd, *J* = 4.0, 2.4, 1.2 Hz)	4′′	6.96 (s, 1H, CH)
	4	6.36 (ddd, *J* = 4.0, 2.4, 2.4 Hz)	6′′	2.39 (t, 2H, *J* = 7.6 Hz)
	5	7.23 (ddd, *J* = 2.4, 1.6, 1.2 Hz)	7′′	1.53 (quint, 2H, *J*= 7.6 Hz)
	1′	12.71 (s, 1H, NH)	8′′ & 9′′	1.37–1.21 (m, 4H)
	4′	6.08 (d, 1H, *J* = 2.0 Hz, CH)	10′′	0.89 (s, 3H, *J* = 7.6 Hz, Me)
	7′	4.01 (s, 3H, OMe)	11′′	2.54 (s, 3H, Me)
**^13^C-NMR**	**Carbon Number**	**Prodigiosin Carbons ppm**	**Carbon Number**	**Prodigiosin Carbons ppm**
	2	122.3	2′′	128.5
	3	117.0	3′′	147.1
	4	111.7	4′′	116.0
	5	127.0	5′′	125.2
	2′	120.7	6′′	25.3
	3′	165.8	7′′	29.8
	4′	92.8	8′′	31.4
	5′	147.7	9′′	22.5
	7′	58.7	10′′	14.3
	8′	128.4	11′′	12.5

## Data Availability

Data is contained within the article or supplementary material.

## Supplementary Materials

The following are available online at https://www.mdpi.com/article/10.3390/molecules27186030/s1, Figure S1A–1B: Extraction of prodigiosin, Figure S2: Absorption spectrum of acetone-extracted prodigiosin, Figure S3: ^13^C CRAPT NMR (CDCl_3_) spectrum of prodigiosin, Figure S4: ^13^C CRAPT NMR (CDCl_3_) spectrum of prodigiosin (processed with resolution booster and auto-cut spectrum), Figure S5: ^1^H NMR (CDCl_3_) spectrum of prodigiosin, Figure S6: ^1^H-^13^C-gHSQC NMR (CDCl_3_) spectrum of prodigiosin, Figure S7A–7D: ^1^H-^1^H-gDQFCOSY NMR (CDCl_3_) spectrum of prodigiosin. Figure S8: ^1^H-^1^H-ROESYAD NMR (CDCl_3_) spectrum of prodigiosin, Figure S9A–9E: ^1^H-^13^C-gHMBC NMR (CDCl_3_) spectrum of prodigiosin, Figure S10A–10K: ^1^H-^1^H-Homonuclear decoupled NMR spectrum of (CDCl3) spectrum of prodigiosin.

## Sample Availability

Samples of the compounds are available from the authors.
