# Supplementary material for: Extraction of the Anticancer and Antimicrobial Agent, Prodigiosin, from Vibrio gazogenes PB1 and Its Identification by 1D and 2D NMR
Source: Molecules. 2022 Sep 16;27(18):6030. doi: 10.3390/molecules27186030 (PMC9504601; doi:10.3390/molecules27186030)
Supplement: Supplementary file 1 [file molecules-27-06030-s001.zip › molecules-1896121-supplementary.pdf]

Supplementary Material

Extraction of the anticancer and antimicrobial agent, prodigiosin, from *Vibrio gazogenes* PB1 and its identification by 1D and 2D NMR

***Dhanya Vijay*[1], *Nassra S Alshamsi* [1], *Ziad Moussa*[1*], *M. Kalim Akhtar*[1*]**

Affiliations:

[1]Department of Chemistry, College of Science, United Arab Emirates University, Al Ain P.O. Box 15551, United Arab Emirates

Correspondence:

E-mails: zmoussa@uaeu.ac.ae (Z.M.); mk.akhtar@uaeu.ac.ae (M.K.A.)

Content

Methodology. Experimental details for the NMR analyses

Figure S1. Extraction of prodigiosin.

Figure S2. Absorption spectrum of acetone-extracted prodigiosin

Figure S3. ^{13}C CRAPT NMR ($CDCl_3$) spectrum of prodigiosin

Figure S4. ^{13}C CRAPT NMR ($CDCl_3$) spectrum of prodigiosin (processed with resolution booster and auto-cut spectrum)

Figure S5. ^{1}H NMR ($CDCl_3$) spectrum of prodigiosin

Figure S6. ^{1}H-^{13}C-gHSQC NMR ($CDCl_3$) spectrum of prodigiosin

Figure S7. ^{1}H-^{1}H-gDQFCOSY NMR ($CDCl_3$) spectrum of prodigiosin

Figure S8. ^{1}H-^{1}H-ROESYAD NMR ($CDCl_3$) spectrum of prodigiosin

Figure S9. ^{1}H-^{13}C-HMBC NMR ($CDCl_3$) spectrum of prodigiosin

Figure S10. ^{1}H-^{1}H-Homonuclear decoupled NMR ($CDCl_3$) spectrum of prodigiosin

Methodology. Experimental details for the NMR analyses

Ref. sample TMS, 1% in $CDCl_3$

1. <u>^{1}H NMR of prodigiosin:</u>
   Spectra width: -2.0-14.0 ppm (6410.3 Hz)
   Number of scans: 128
   Relaxation delay: 1s
   Pulse angle: 45 degrees
   Receiver gain (dB): 6.0
   Complex points: 16384
   Block size: 32
   Observe pulse: 4.40 µs
   Calibration pw90: 8.80 µs at power 61 dB
   Nucleus frequency: H1 = 399.732 MHz
   Decouple C13 frequency 100.522 MHz

2. <u>^{13}C CRAPT NMR of prodigiosin:</u>
   Spectra width: -14.4-234.4 ppm (25000 Hz)
   Number of scans: 10000
   Relaxation delay: 1s
   Pulse angle: 45 degrees
   Receiver gain (dB): 5.0
   Complex points: 32768
   Block size: 64
   Observe pulse: 4.58 µs
   Calibration pw90: 9.15 µs at power 55 Db
   Pulse sequence: CRISIS with 1999.2 µs width and 40 dB of power; One-bond XH coupling constant used is 146 Hz.
   HiBand-inversion: BIP; Shape pib 60 us, 60 µs width and 6.2 dB of power
   Observe channels: C13 frequency 100.523 MHz; Offset 1530.50 Hz; 90 degree at power 9.15 µs at 55.
   Delay 0.5 s before starting acquisition

3. <u>gHSQC 2D NMR of prodigiosin:</u>
   Acquisition in F1: Spectra width: -10.0-160.0 ppm (17086.7Hz)
   Scans per t1 increment = 4; increments in t1 = 128; resolution = 133 Hz or 1.33 ppm
   T1 increments =128; one-bond J1xh = 146.0 Hz.
   Acquisition in F2: Spectra width: 6410.3 Hz; complex points = 962
   Number of scans: 50
   Receiver gain (dB): 3.0; timing rof2 25.2 µs; alfa 10.0; ddrtc 35.2
   Relaxation delay: 1s
   Observe pulse/at power: 8.8 µs at 61; observe offset 399.70 Hz;
   Gradient selection: strength 1051; time 2.0 ms; E/D ratio 3.9755; recovery time 0.5 ms
   Steady state Grad-90 Grad option: strength = 1260, time = 2 ms
   X-channel-X pulse width 11.1 µs, X pulse power 54 dB, X offset -1987.40 Hz, decoupler power 33, modulation mode ccp, modulation frequency 29412
   Decouple C13 100.520 MHz

4. <u>gHMBC 2D NMR of prodigiosin:</u>
   Acquisition in F1: Spectra width: -15.0-225.0 ppm (24125.5Hz)
   Scans per t1 increment = 4; increments in t1 = 200; resolution = 121 Hz or 1.2 ppm
   T1 increments =200; multiple-bond J1xh = 8.0 Hz.
   Acquisition in F2: Spectra width: 6410.3 Hz; complex points = 962
   Number of scans: 50
   Receiver gain (dB): 30; timing rof2 25.2 µs; alfa 10.0; ddrtc 40.0
   Relaxation delay: 1s
   Observe pulse/at power: 8.8 µs at 61; observe offset 399.70 Hz; Spectra width: 6410.3 Hz;
   Gradient selection: strength 1051; time 2.0 ms; E/D ratio 3.9755; recovery time 0.5 ms
   Steady state Grad-90 Grad option: strength = 1260, time = 2 ms
   One-bond suppression: coupling 1 high 165.0 Hz, coupling 2 low 130.0, G-strength 1050, G-time 1.6 ms
   S6 refocusing pulse 90 deg. Pulse 31.7 µs, power level 43 dB
   X-channel-X pulse width 11.1 µs, X pulse power 54 dB, X offset 1027.90 Hz, decoupler power 33, modulation mode ccc, modulation frequency 29412
   Decouple C13 100.523 MHz.

5. <u>gDQCOSY 2D NMR of prodigiosin:</u>
   Acquisition in F2: Spectra width: 6410.3Hz
   Scans per t1 increment = 1; increments in t1 = 200; complex points 962, resolution = 32 Hz or 0.08 ppm
   T1 increments =200;
   Number of scans: 50
   Receiver gain (dB): 30; timing rof2 25.2 µs; alfa 10.0; ddrtc 40.0
   Relaxation delay: 1s
   Observe pulse/at power: 8.8 µs at 61;
   Gradient selection: strength 841; time 1.0 ms; E/D ratio 0.5; recovery time 0.5 ms
   Steady state Grad-90 Grad option: strength = 1260, time = 2 ms
   observe offset 399.70 Hz; 90 deg. Power at 8.80 µs
   Decouple C13 100.522 MHz.

6. <u>Homodecoupling NMR of prodigiosin:</u>
   Number of scans: 8, presaturate with decoupling duty cycle = 10%.
   Spectra width: 6410.3Hz
   Acquisition time 2.556 s
   Complex points 16384
   Steady state 2
   Block size 32
   Relaxation delay: 1s
   Observe pulse 8.8 µs or 90 deg.
   Calibration pw90 8.80 µs at power 61 dB
   Receiver gain (dB): 30; timing rof2 25.2 µs; alfa 10.0; ddrtc 40.0
   Homo-decoupling offset 399.70 Hz; power 1 dB, duty cycle 10%
   Homo-presaturation Delay 1s, offset 399.7 Hz, power 0 dB
   Homodec Observe H1 channel Dec modulation offset 99.70 Hz, 90 deg. At pwr 8.80 at power 61 dB
   Decouple C13 100.522 MHz.

A

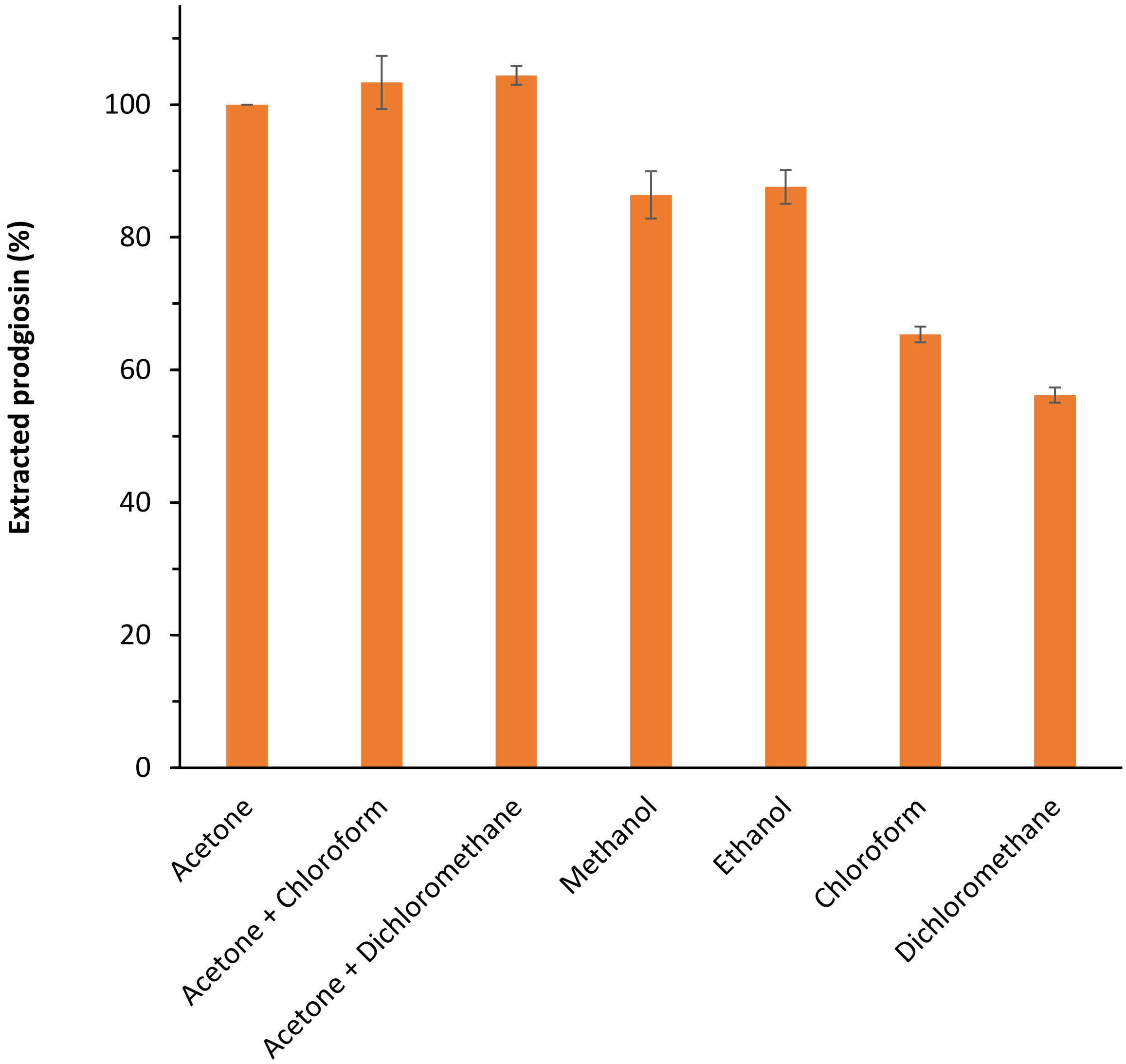

Extracted prodgiosin (%)
0
20
40
60
80
100
Acetone
Acetone + Chloroform
Acetone + Dichloromethane
Methanol
Ethanol
Chloroform
Dichloromethane

B

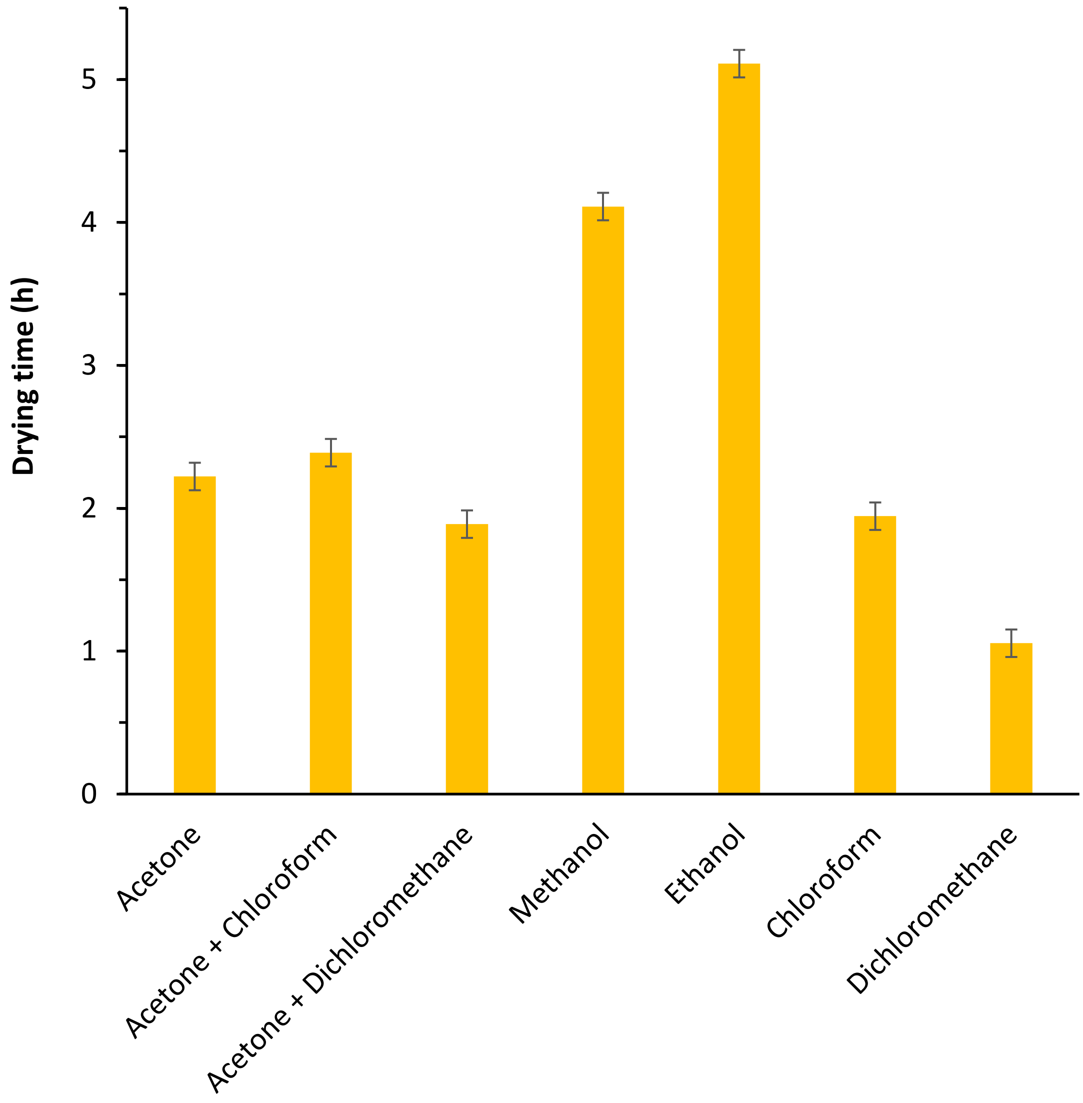

Figure S1. Extraction of prodigiosin. Prodigiosin was extracted from *V. gazogenes* PB1 cells using different organic solvents. (A) The amount of extracted prodigiosin was quantitated by measuring the absorbance at 535nm and expressed as a % relative the amount of prodigiosin extracted with acetone. (B) Samples were left to air-dry at 40°C to determine sample drying times. Results are expressed as the mean ± standard deviation.

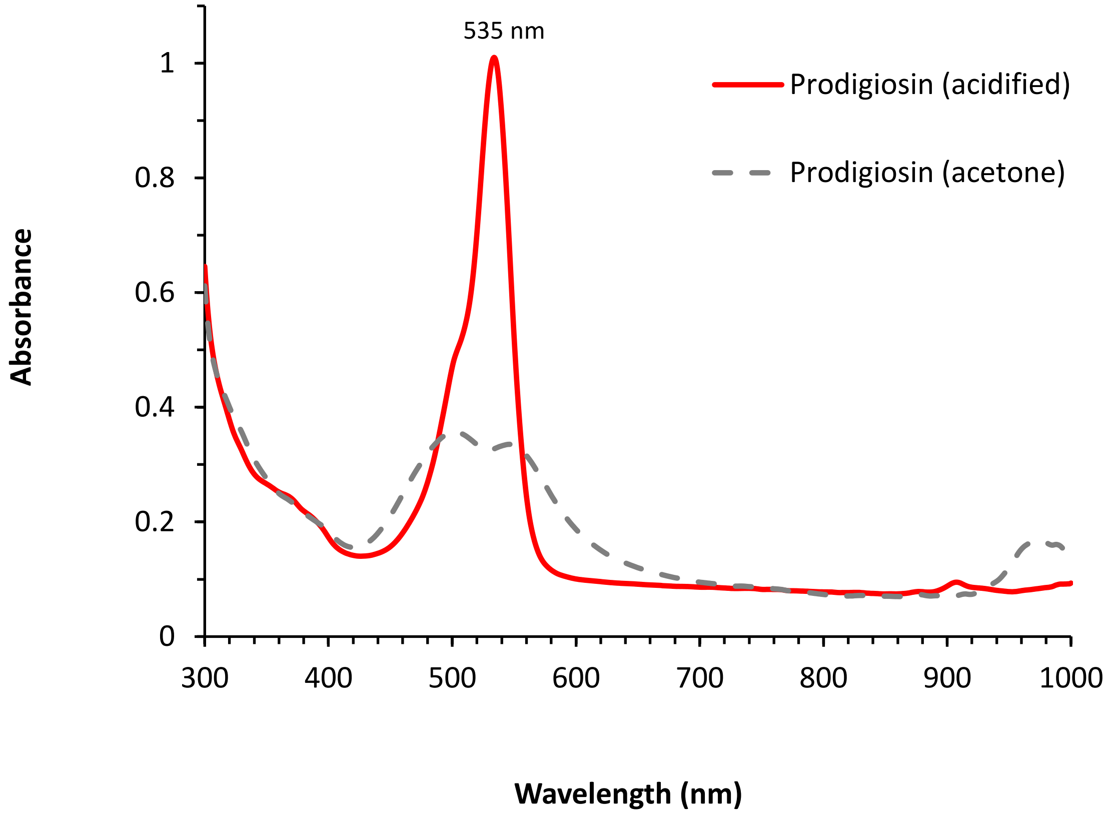

Figure S2. Absorption spectrum of acetone-extracted prodigiosin.

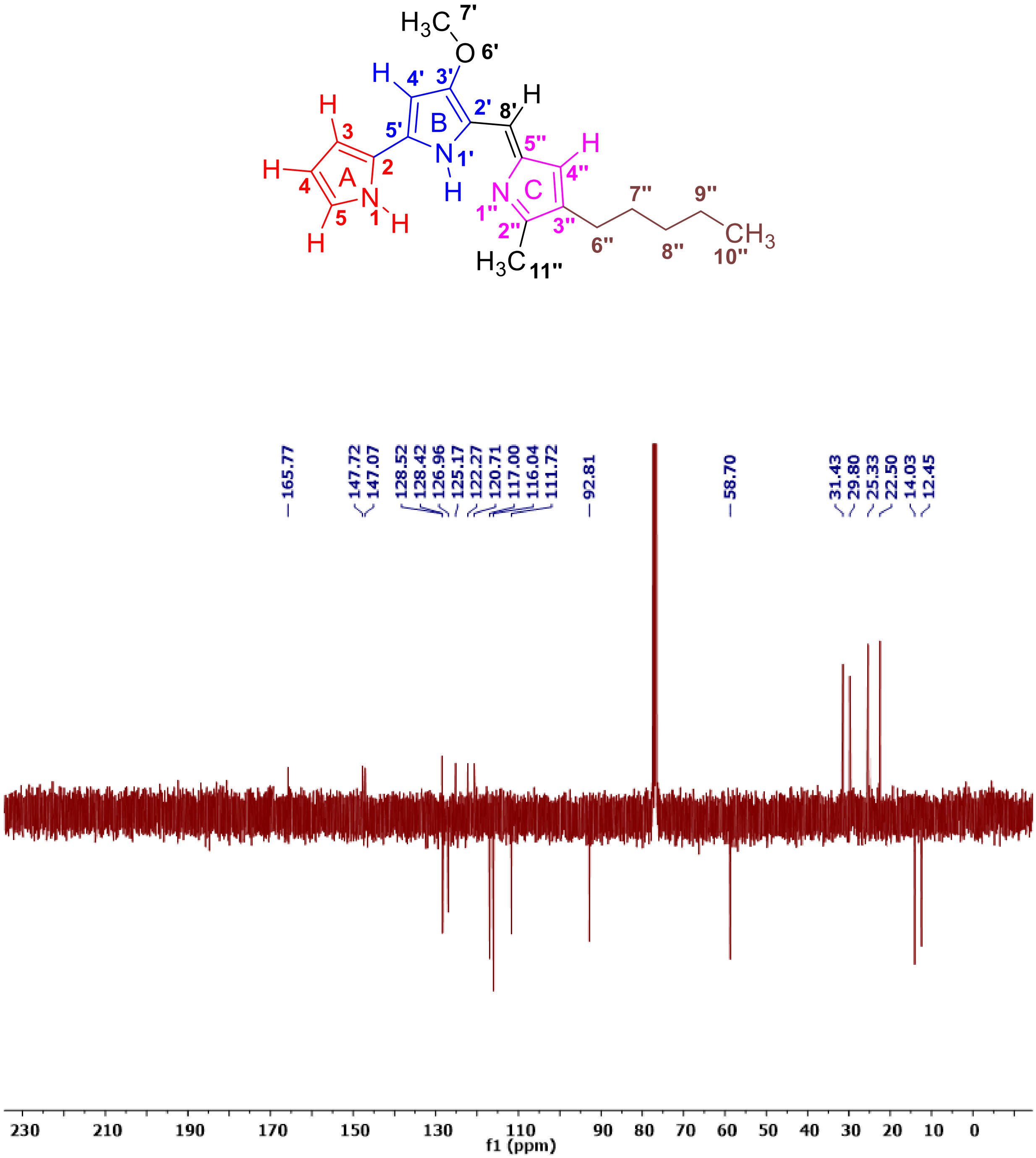

Figure S3. ^{13}C CRAPT NMR ($CDCl_3$) spectrum of prodigiosin

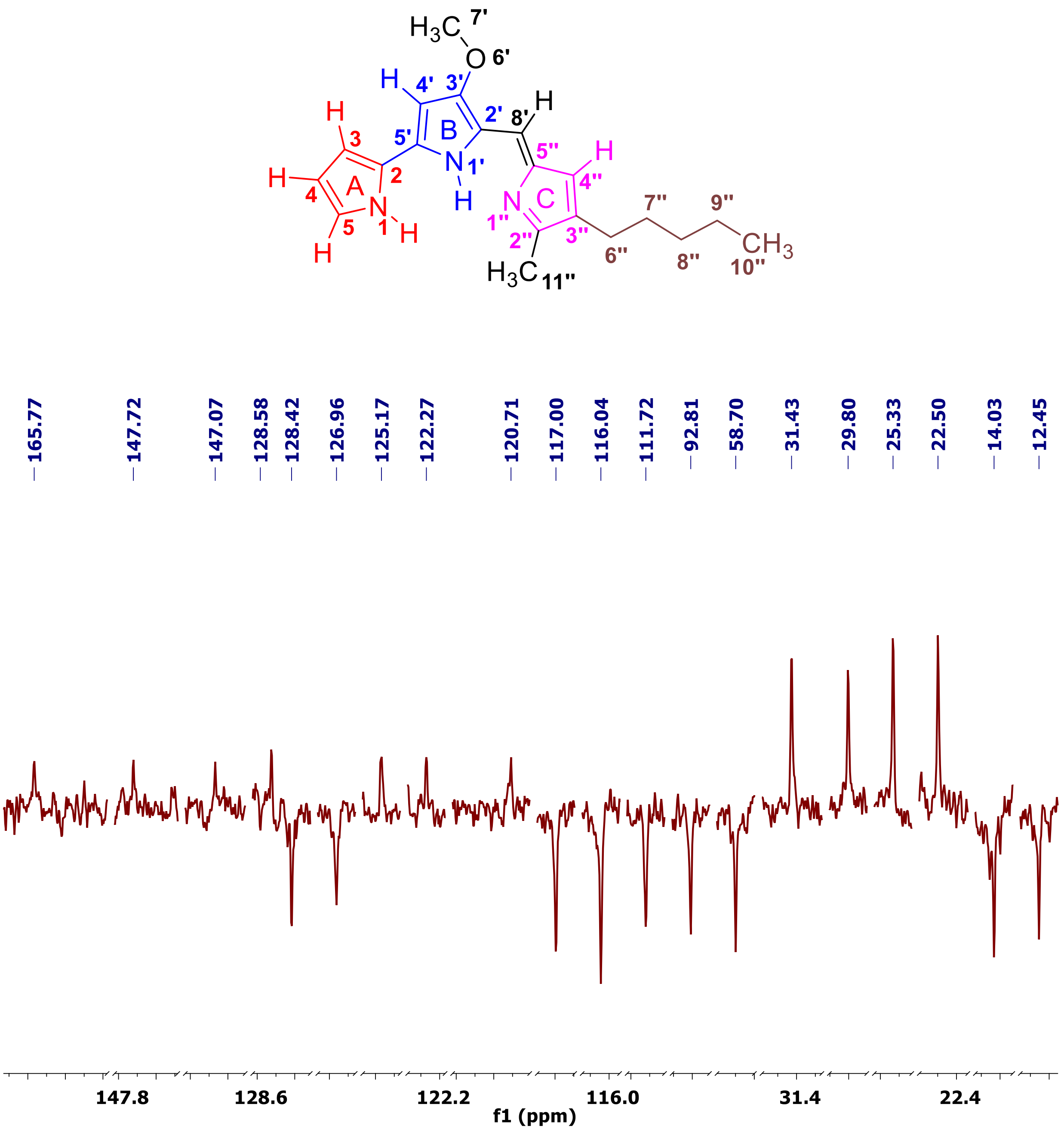

Figure S4. ^{13}C CRAPT NMR ($CDCl_3$) spectrum of prodigiosin (processed with resolution booster and auto-cut spectrum)

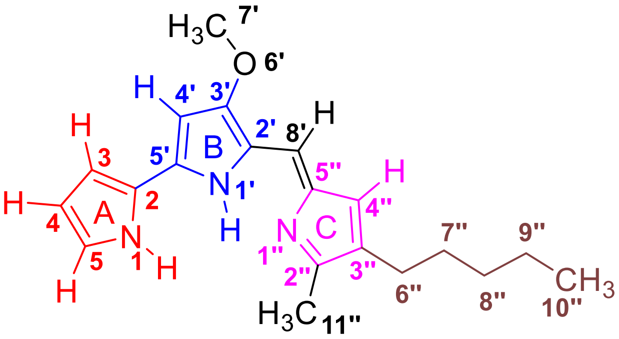

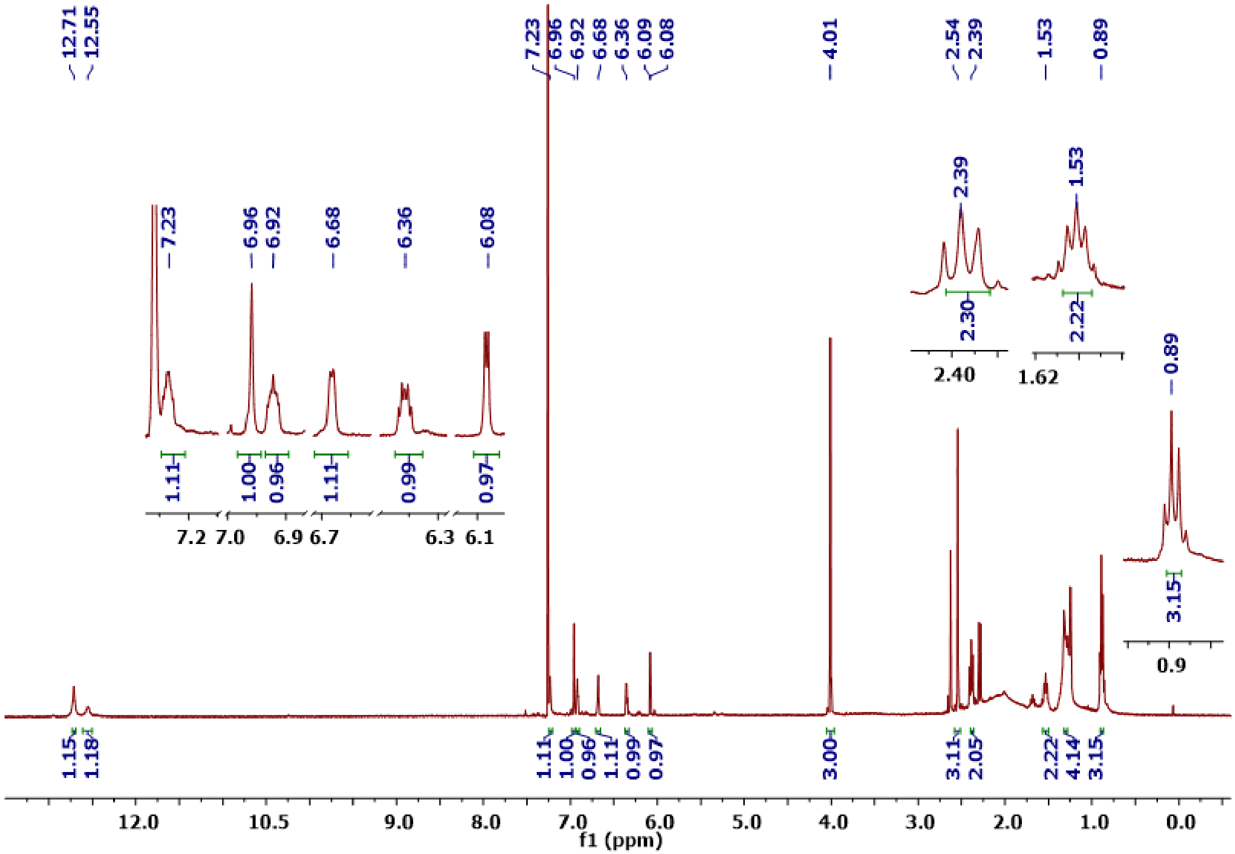

Figure S5. ^{1}H NMR ($CDCl_3$) spectrum of prodigiosin

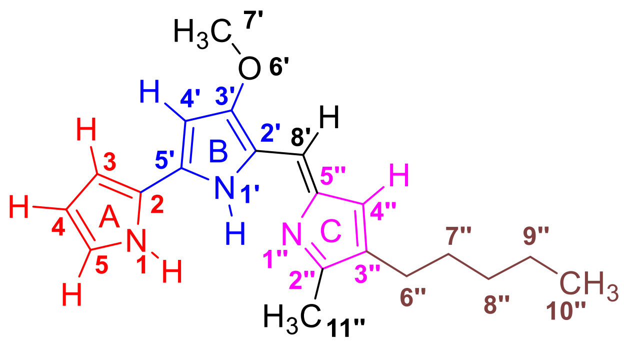

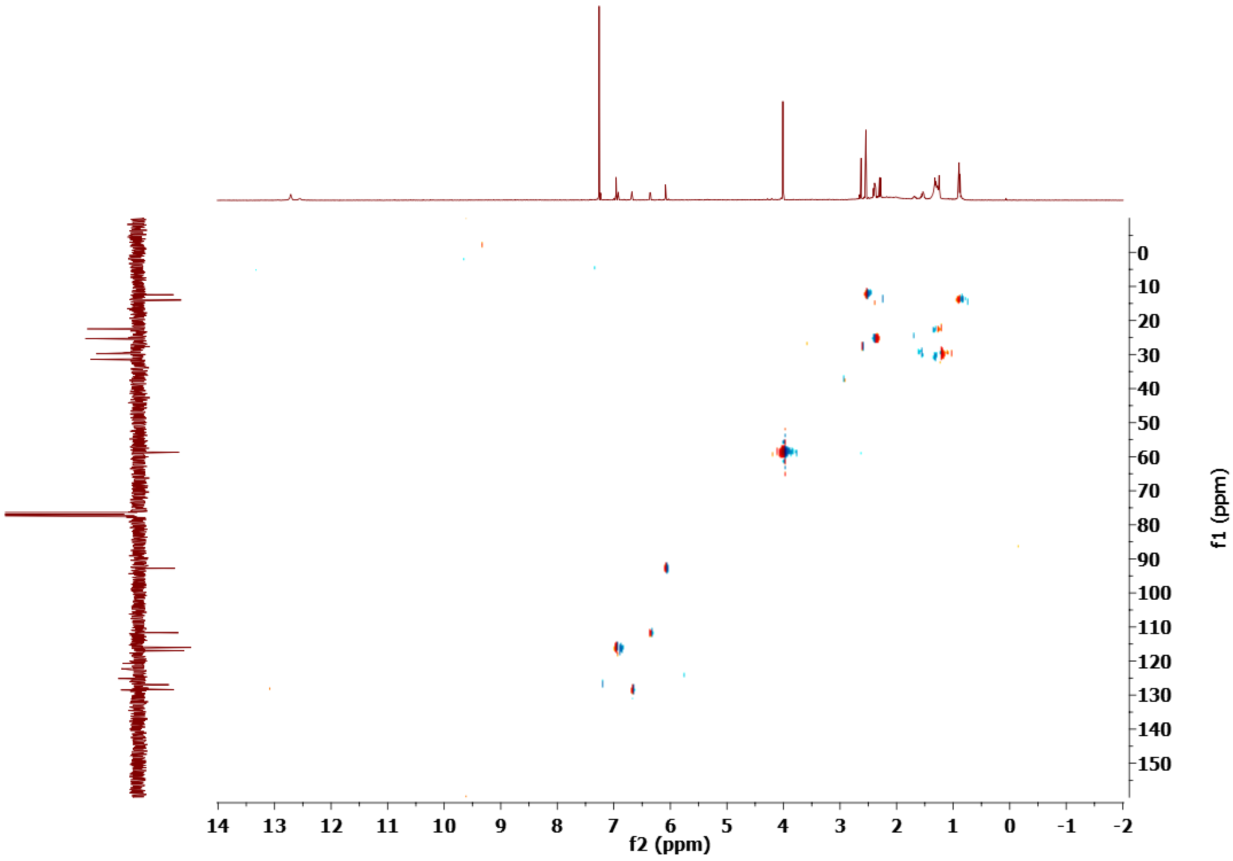

Figure S6. ^{1}H-^{13}C-gHSQC NMR ($CDCl_3$) spectrum of prodigiosin

A

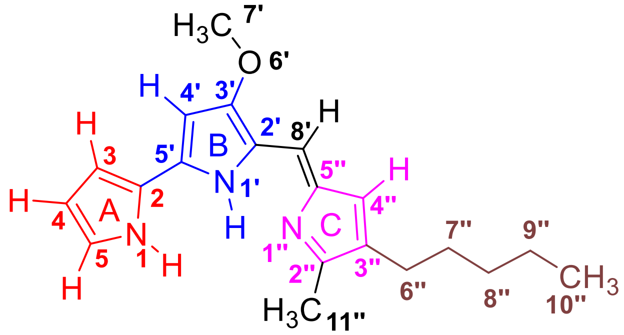

H$_3$C
7'
O
6'
H
4'
3'
2'
8'
H
H
3
5'
B
5''
H
H
4
A
2
N
1'
4''
7''
9''
N
C
1
H
1''
5
H
2''
3''
CH$_3$
H
6''
8''
10''
H$_3$C
11''

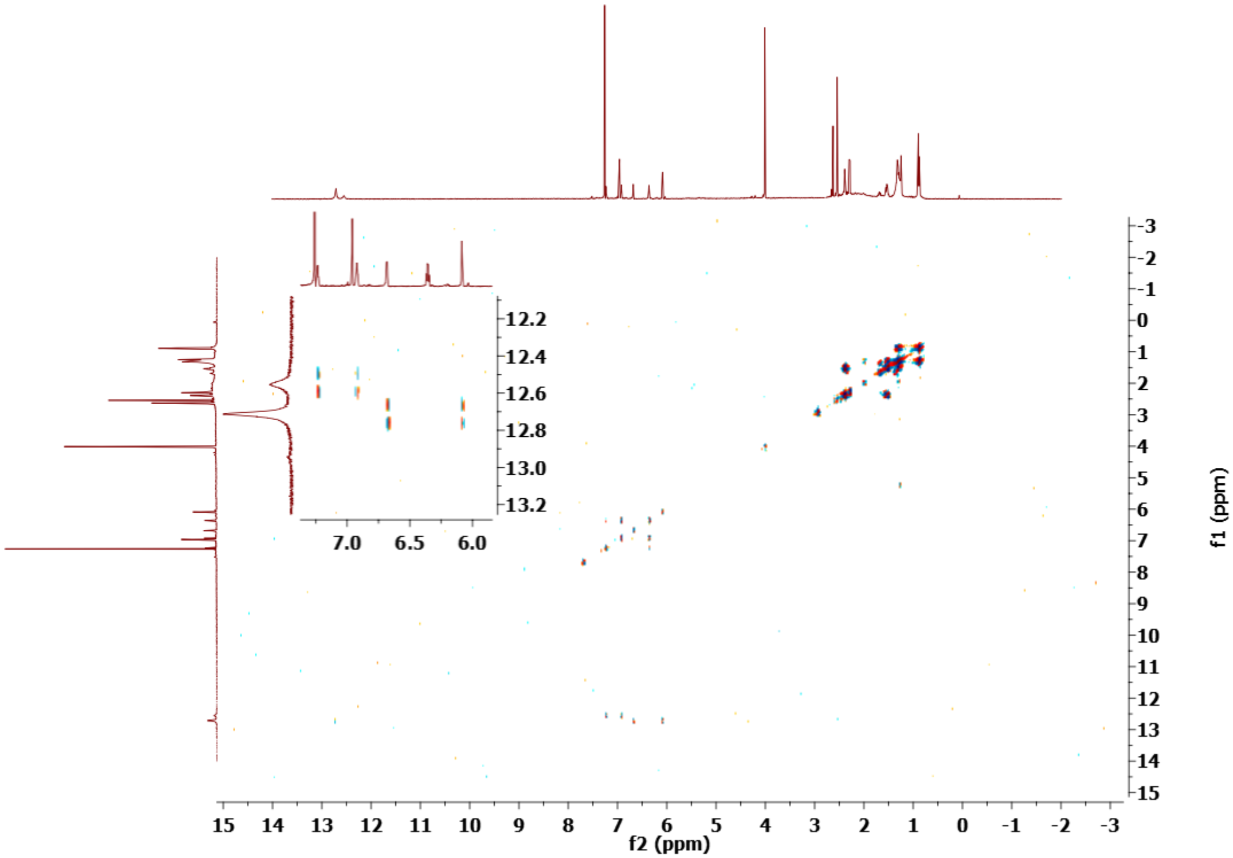

f1 (ppm)
f2 (ppm)

B

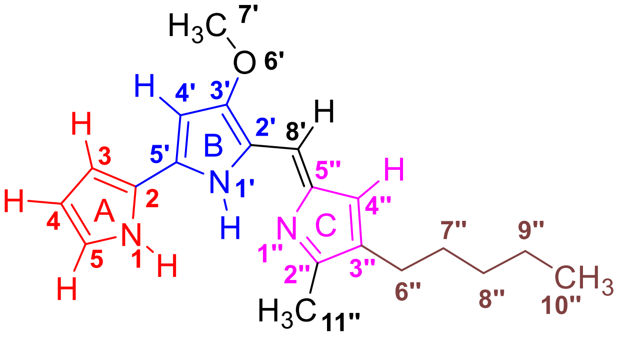
H3C
7'
O
6'
H
4'
3'
2'
8'
H
3
5'
B
N
1'
H
5''
H
4''
H
2
4
A
N
H
C
N
7''
9''
1''
2''
3''
CH3
5
1
H
H
6''
8''
10''
H3C
11''

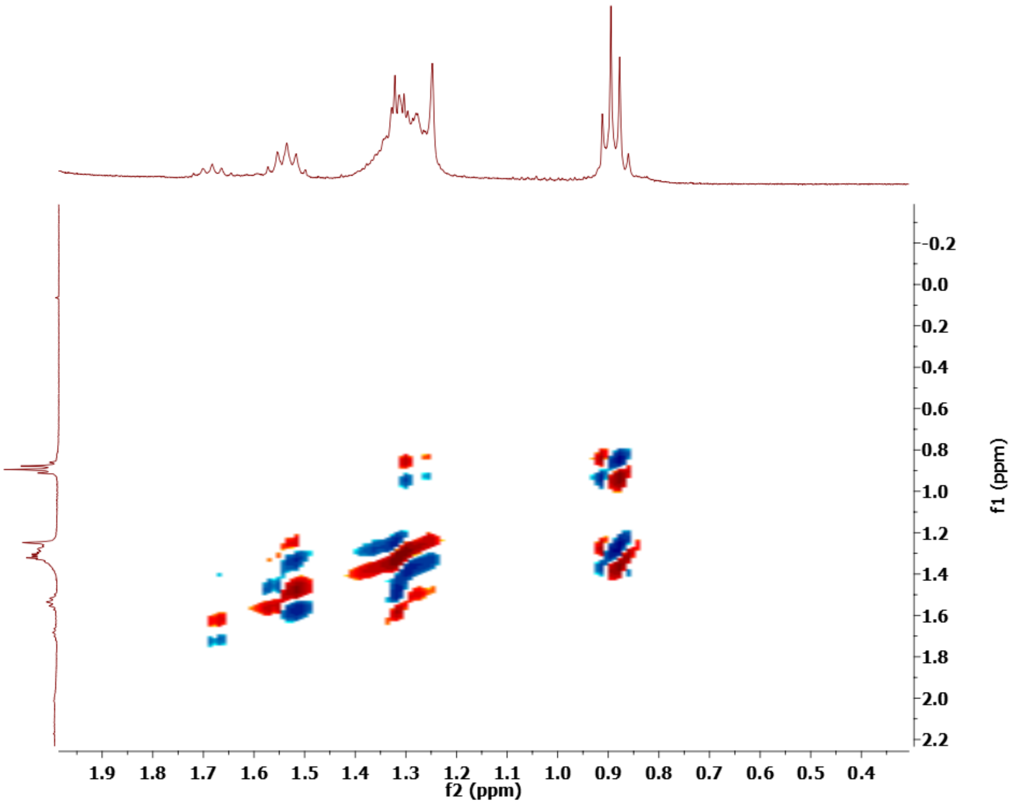
-0.2
0.0
0.2
0.4
0.6
0.8
1.0
1.2
1.4
1.6
1.8
2.0
2.2
f1 (ppm)
1.9 1.8 1.7 1.6 1.5 1.4 1.3 1.2 1.1 1.0 0.9 0.8 0.7 0.6 0.5 0.4
f2 (ppm)

C

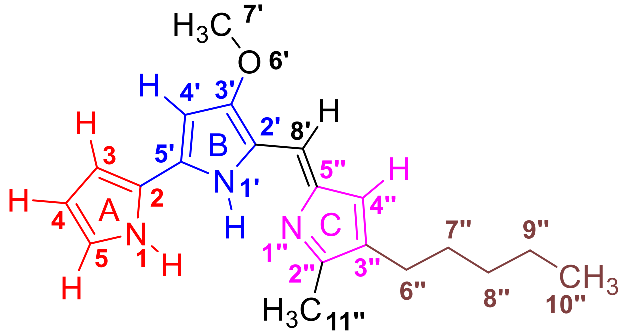

H3C
7'
O
6'
H
4'
3'
2'
8'
H
H
3
5'
B
N
1'
H
2
A
N
1
H
4
5
H
H
1''
N
C
2''
3''
5''
4''
H
6''
7''
8''
9''
CH3
10''
H3C
11''

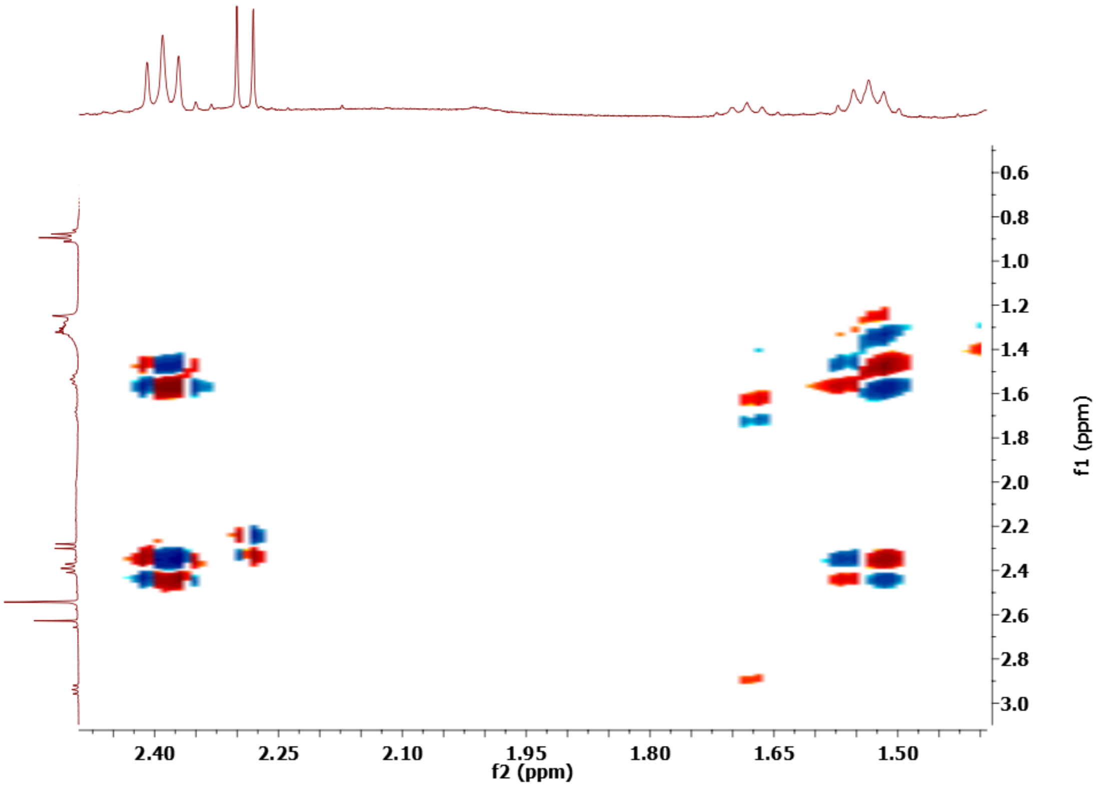

f1 (ppm)
f2 (ppm)

D

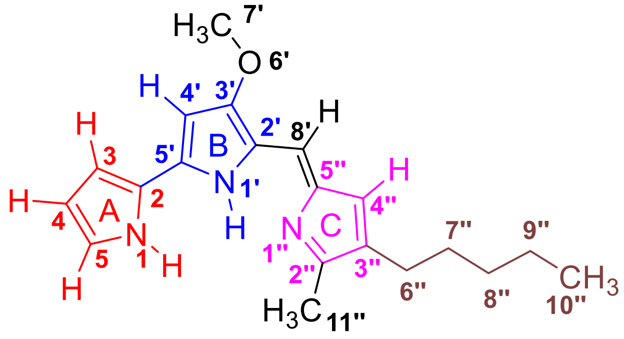

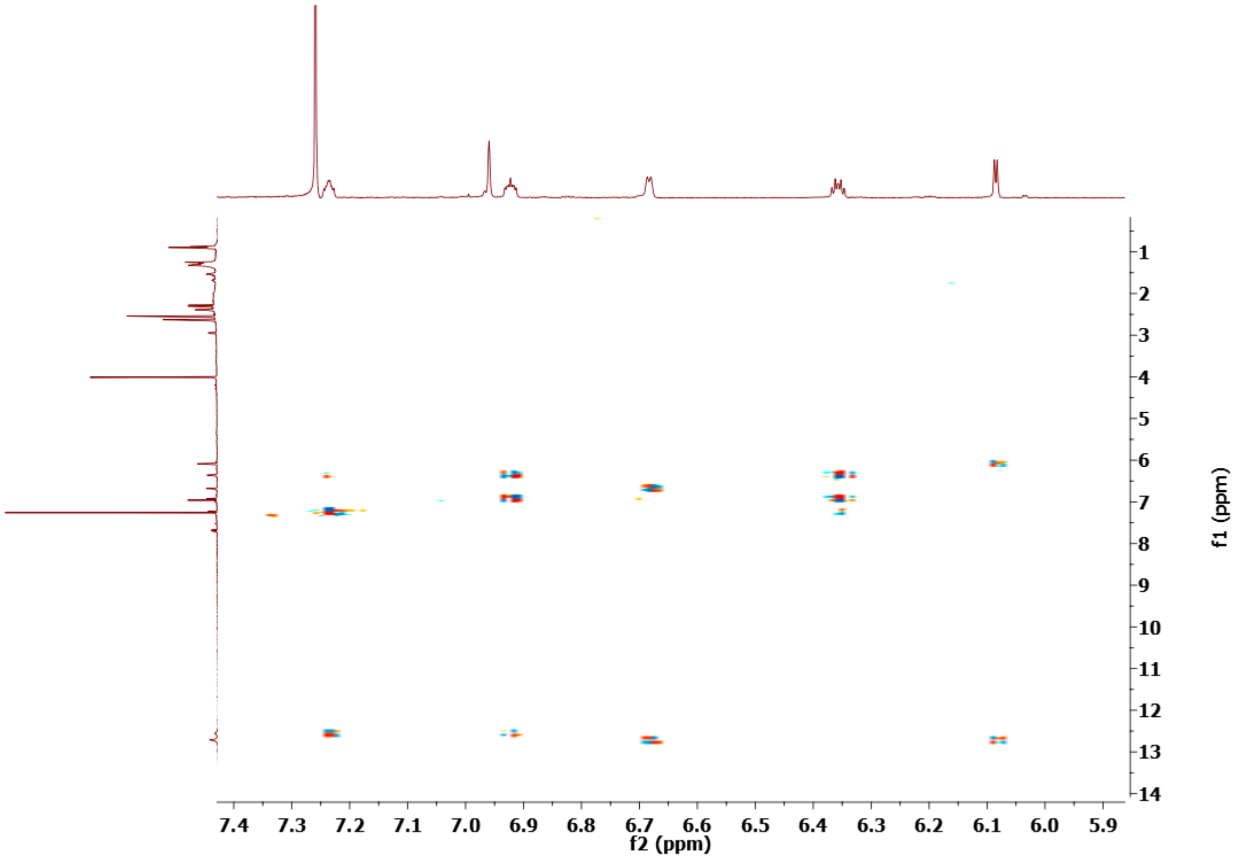

Figure S7. ^{1}H-^{1}H-gDQFCOSY NMR ($CDCl_3$) spectrum of prodigiosin. (**A**) Full spectrum, (**B**) Part of the spectrum showing correlations between C7'', C8'', C9'', and C10'', (**C**) Part of the spectrum showing correlations between C6'', and C7'', (**D**) Part of the spectrum showing correlations between the NH's and pyrrolidine protons.

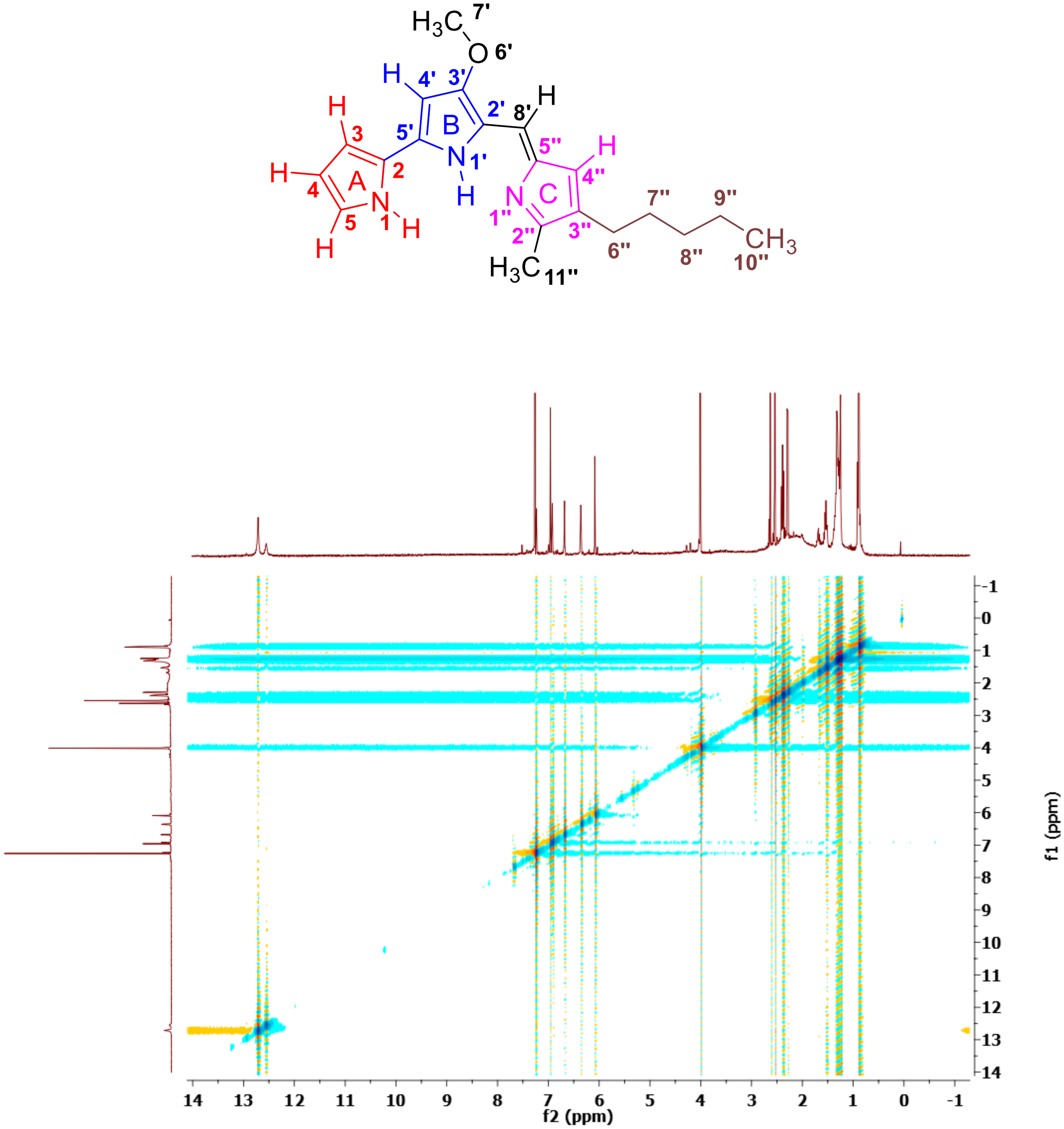

Figure S8. ^{1}H-^{1}H-ROESYAD NMR ($CDCl_3$) spectrum of prodigiosin

A

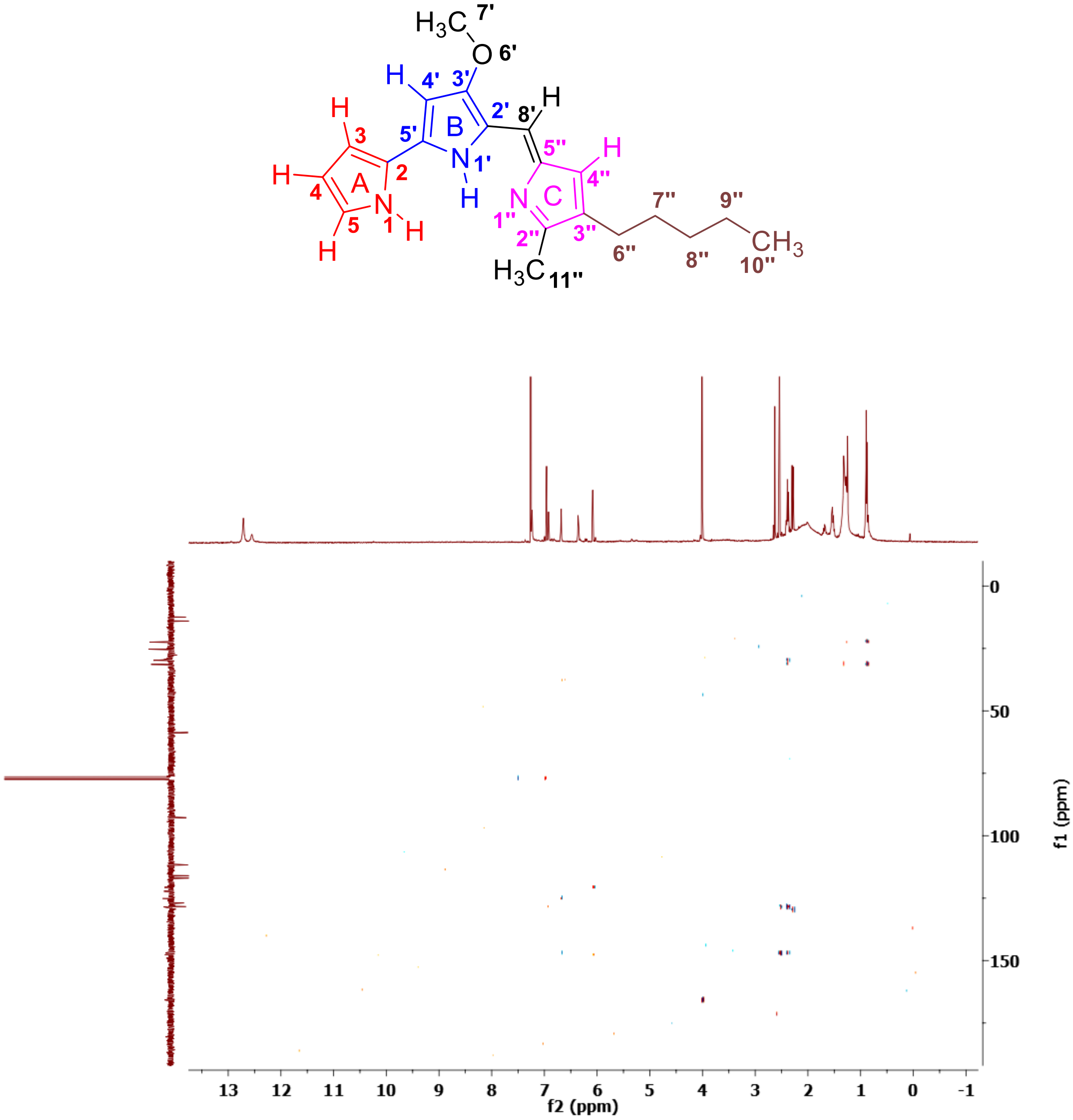

H$_3$C 7'
O 6'
H 4' 3'
2' 8' H
H 3
5' B
N 1'
H
5'' H
H
2
4 A
N
4''
7'' 9''
N C
1'' 2'' 3''
CH$_3$
5 1 H
H
6'' 8'' 10''
H$_3$C 11''
f1 (ppm)
f2 (ppm)

B

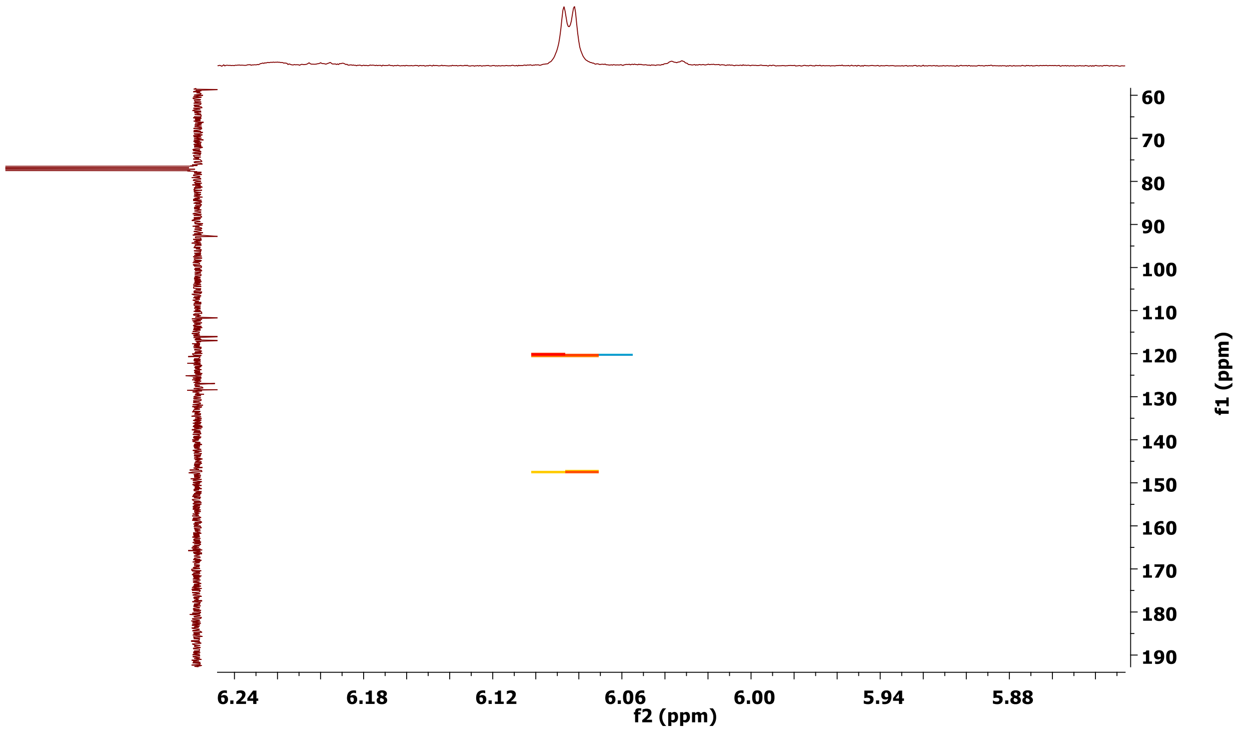

60
70
80
90
100
110
120
130
140
150
160
170
180
190
f1 (ppm)
6.24
6.18
6.12
6.06
6.00
5.94
5.88
f2 (ppm)

C

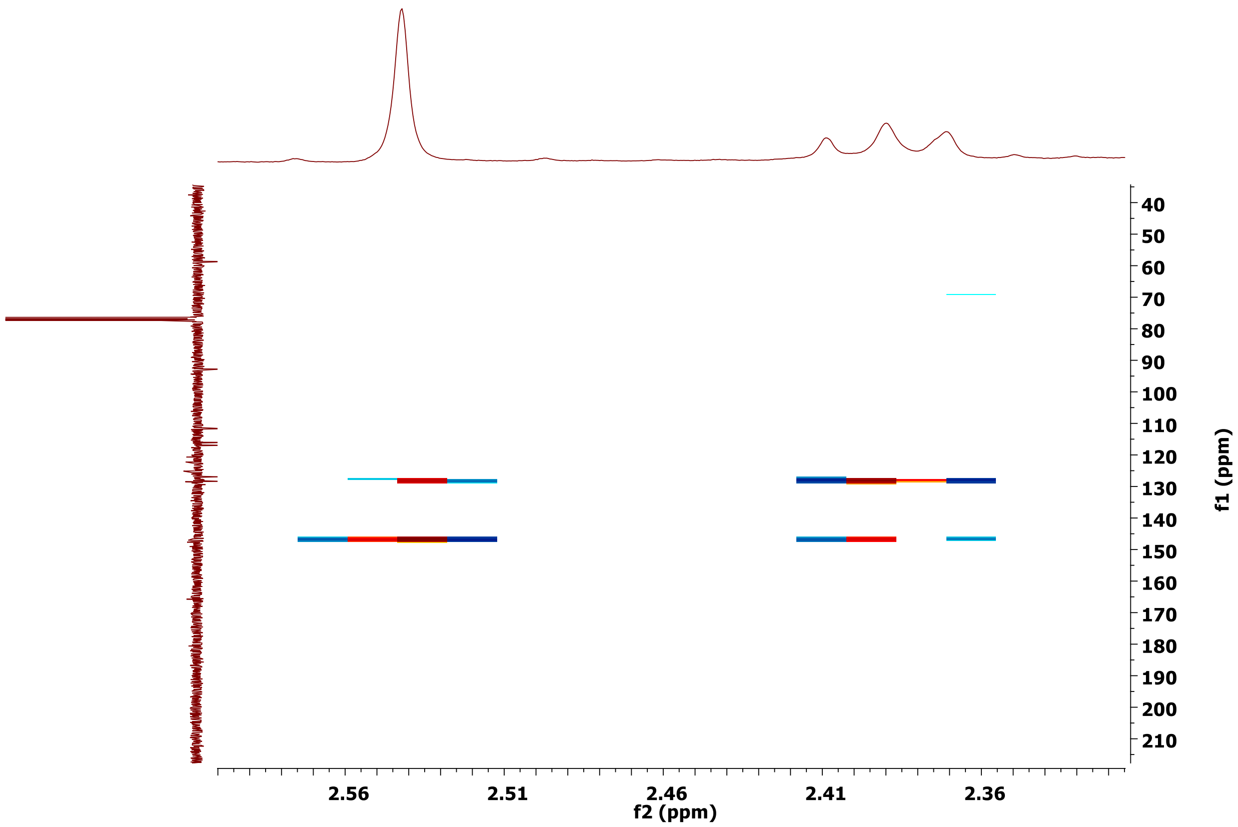

40
50
60
70
80
90
100
110
120
130
140
150
160
170
180
190
200
210
f1 (ppm)
2.56
2.51
2.46
2.41
2.36
f2 (ppm)

D

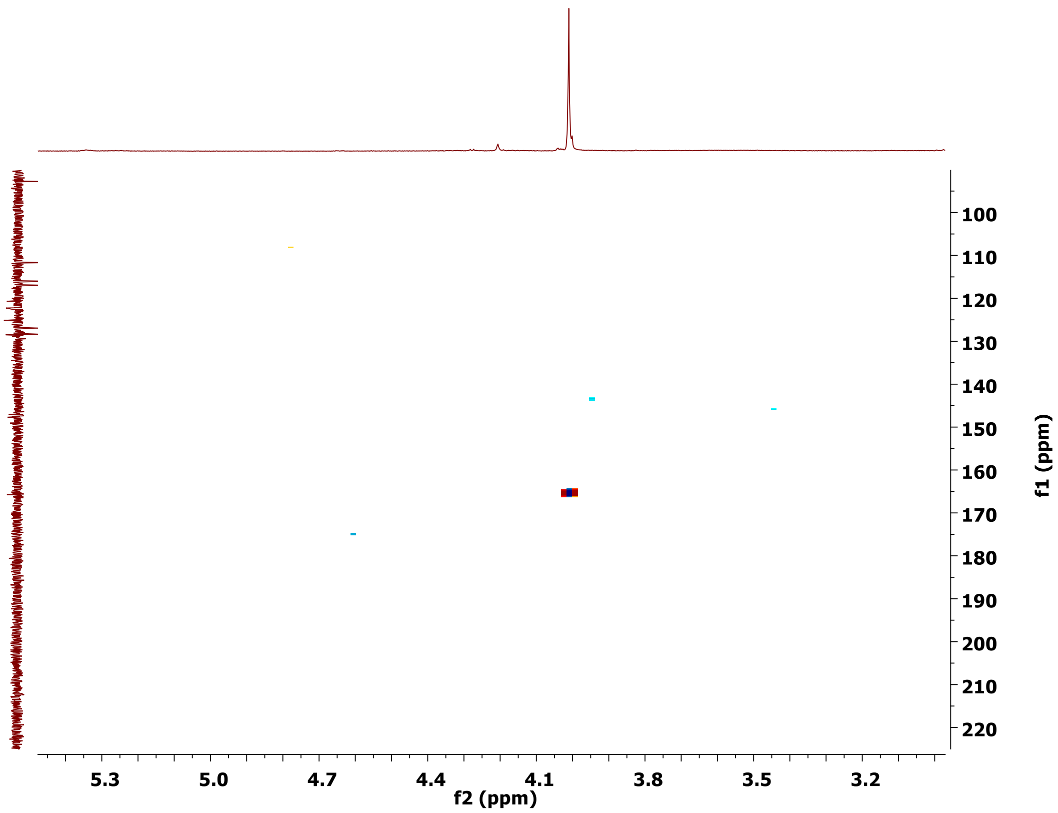

100
110
120
130
140
150
160
170
180
190
200
210
220
f1 (ppm)
5.3
5.0
4.7
4.4
4.1
3.8
3.5
3.2
f2 (ppm)

E

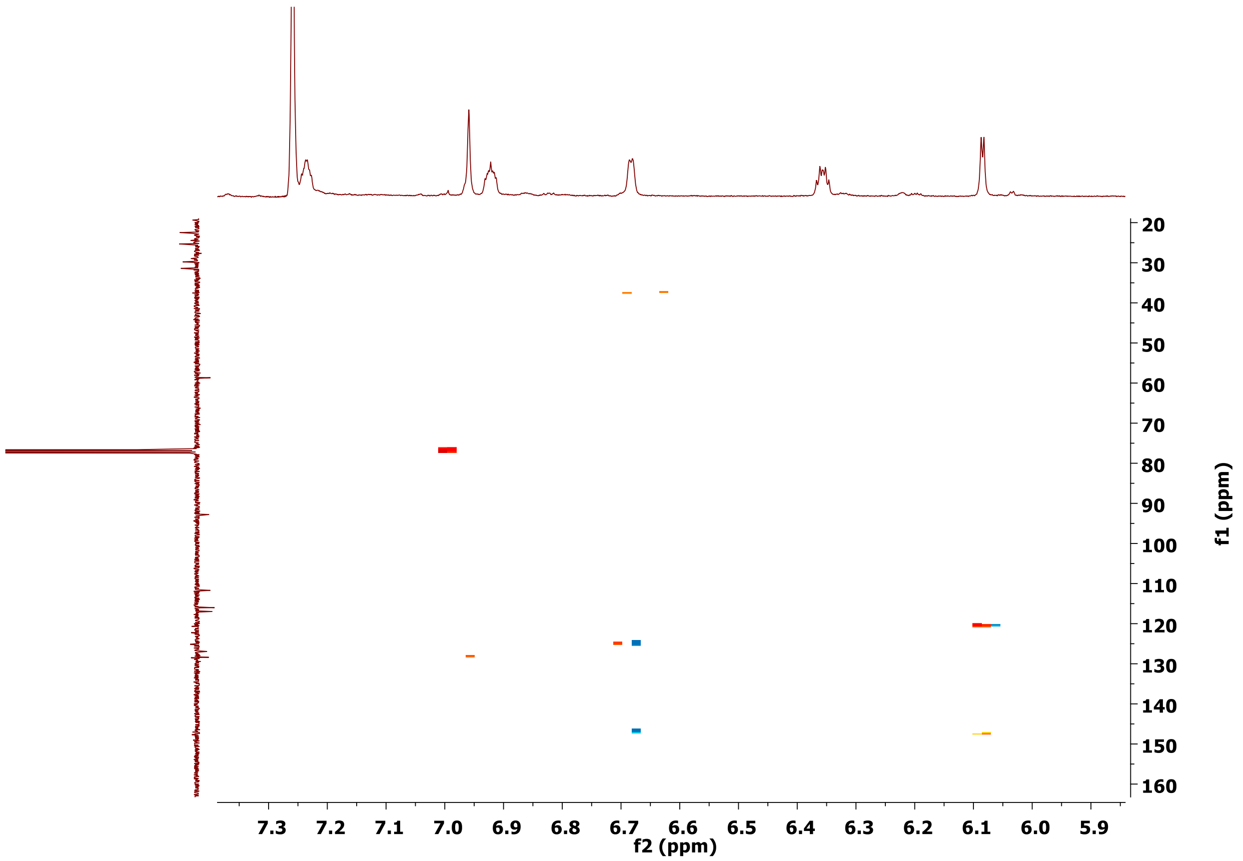

Figure S9. ${}^{1}H$-${}^{13}C$-gHMBC NMR ($CDCl_3$) spectrum of prodigiosin. (**A**) Full spectrum, (**B**) Part of the spectrum showing correlations between $C_{4'}$-H, $C_{2'}$ and $C_{5'}$, (**C**) Part of the spectrum showing correlations between $C_{11''}$-H_3 and $C_{6''}$-H_2 with $C_{3''}$ and $C_{2''}$, (**D**) Part of the spectrum showing correlations between $C_{7'}$-H_3 and $C_{3'}$, (**E**) Part of the spectrum showing various correlations in the aromatic region.

A

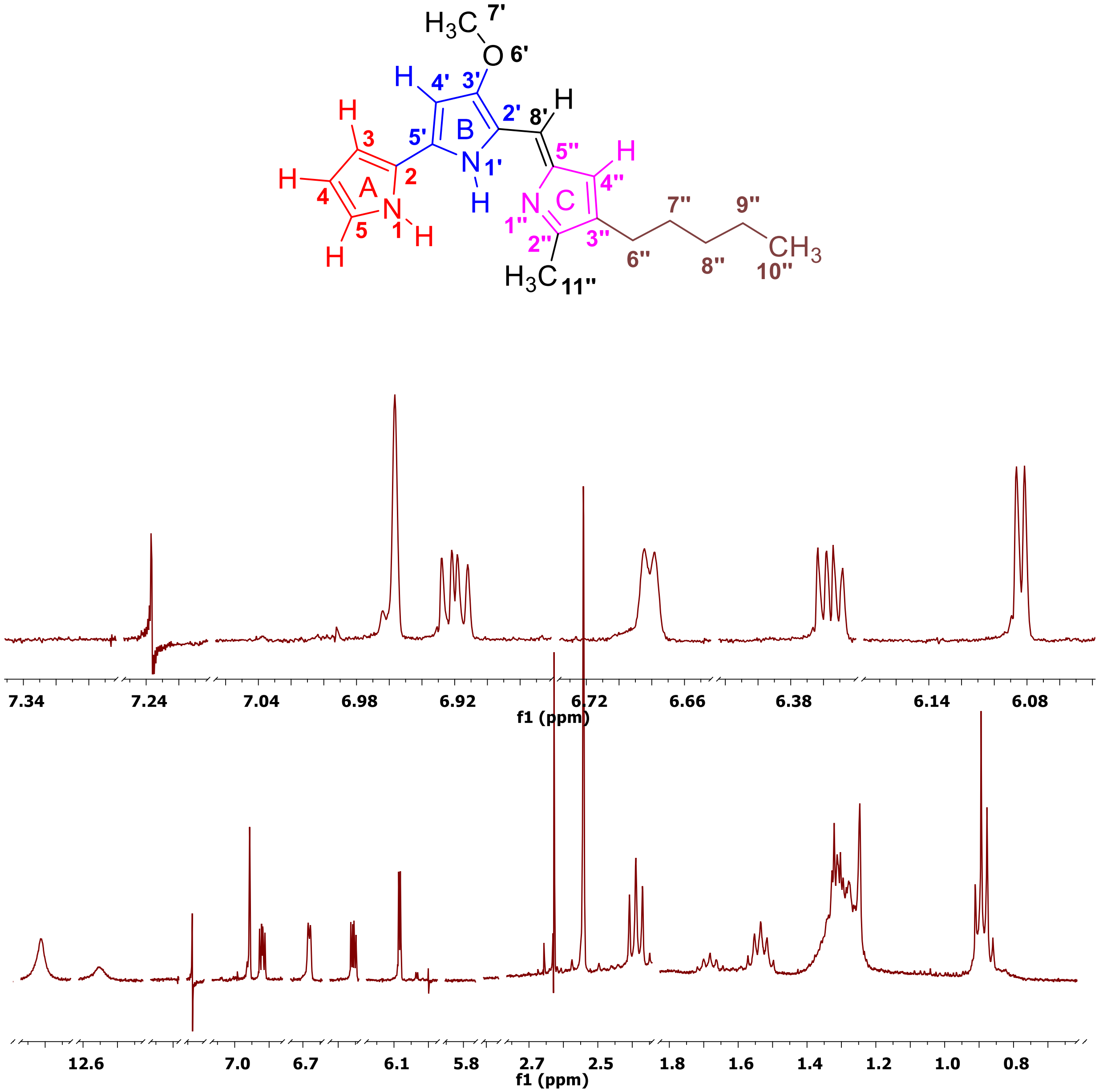
A
B
C
7'
6'
3'
4'
2'
8'
5'
1'
5''
4''
3''
2''
1''
6''
7''
8''
9''
10''
11''
3
2
4
5
1
7.34
7.24
7.04
6.98
6.92
6.72
6.66
6.38
6.14
6.08
f1 (ppm)
12.6
7.0
6.7
6.1
5.8
2.7
2.5
1.8
1.6
1.4
1.2
1.0
0.8
f1 (ppm)

B

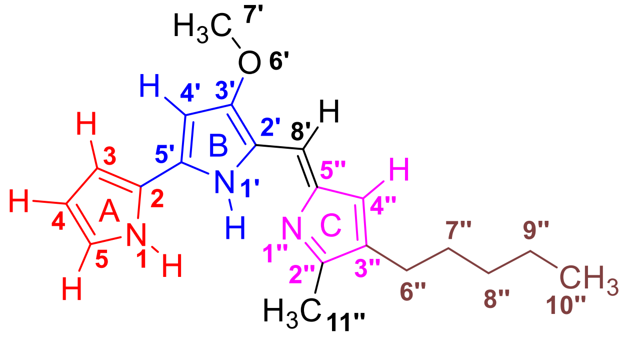
H3C 7'
O 6'
H 4' 3'
2' 8' H
H 3 5' B
H 4 A 2 N 1'
5 N 1 H
H
N C 5'' H 4''
1'' 2'' 3''
7'' 9''
6'' 8'' CH3 10''
H3C 11''

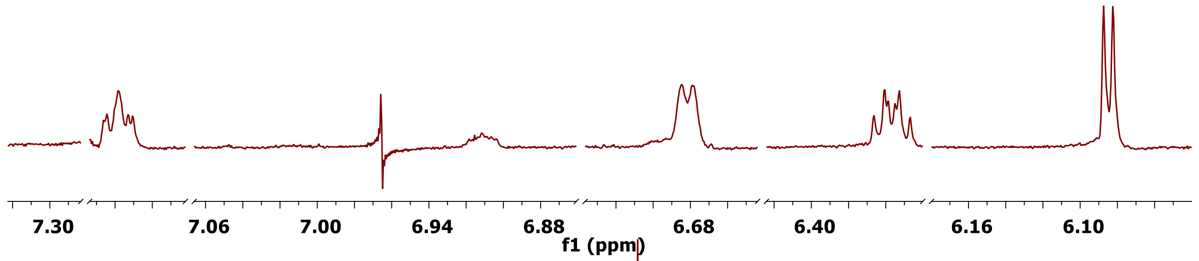
7.30
7.06
7.00
6.94
6.88
6.68
6.40
6.16
6.10
f1 (ppm)

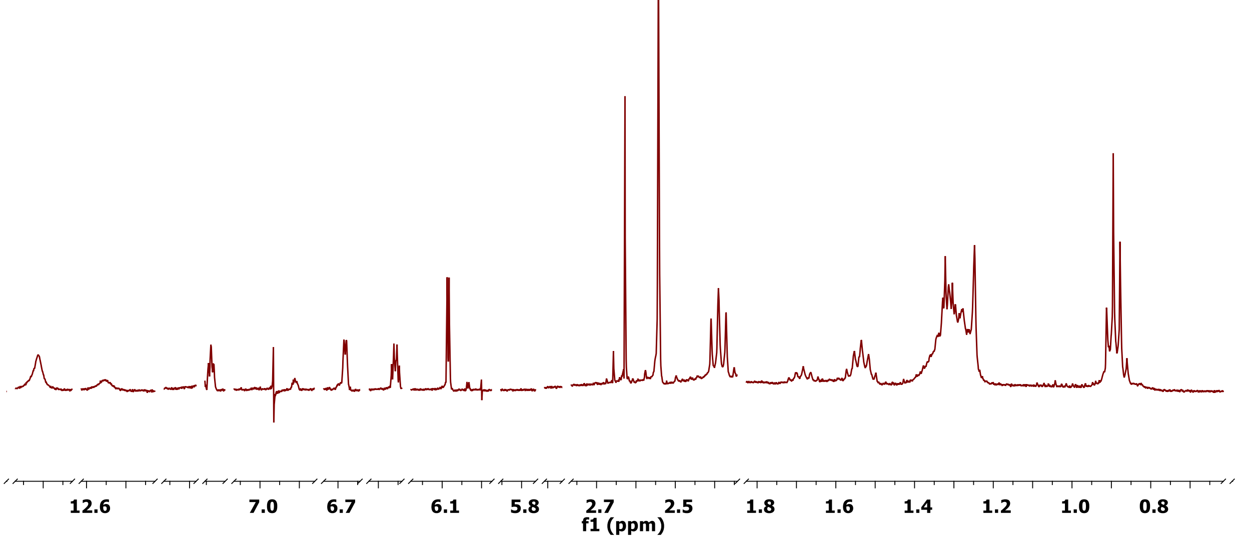
12.6
7.0
6.7
6.1
5.8
2.7
2.5
1.8
1.6
1.4
1.2
1.0
0.8
f1 (ppm)

C

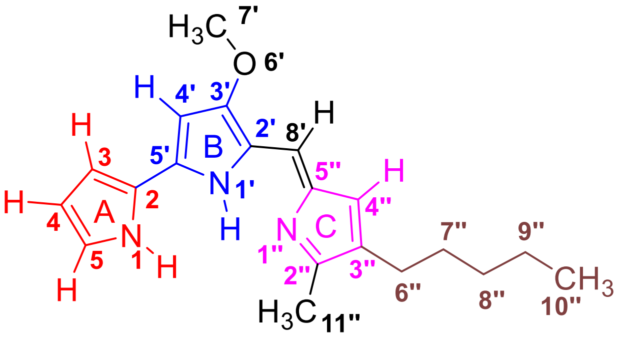
H3C
7'
O
6'
H
4'
3'
2'
8'
B
5'
H
3
H
4
A
2
N
1'
N
1
H
5
H
H
C
5''
4''
1''
2''
3''
7''
9''
6''
8''
CH3
10''
H3C
11''

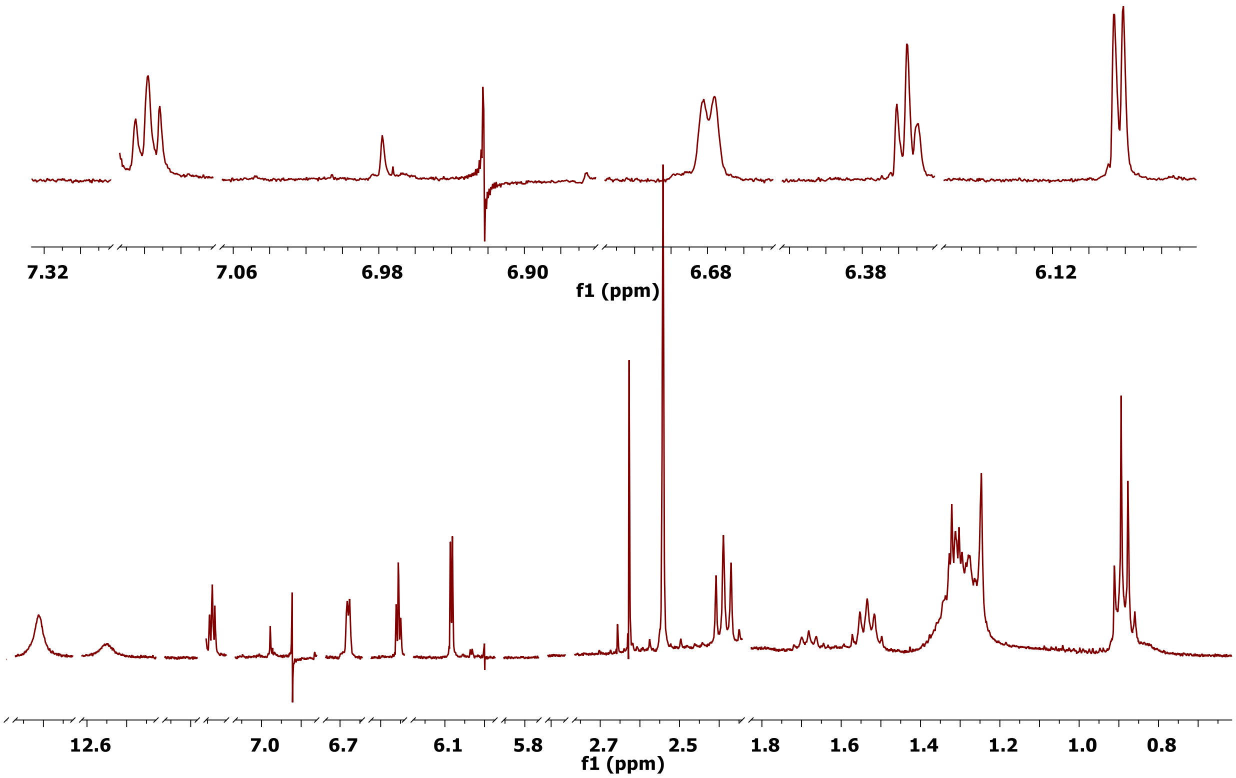
7.32
7.06
6.98
6.90
6.68
6.38
6.12
f1 (ppm)
12.6
7.0
6.7
6.1
5.8
2.7
2.5
1.8
1.6
1.4
1.2
1.0
0.8
f1 (ppm)

D

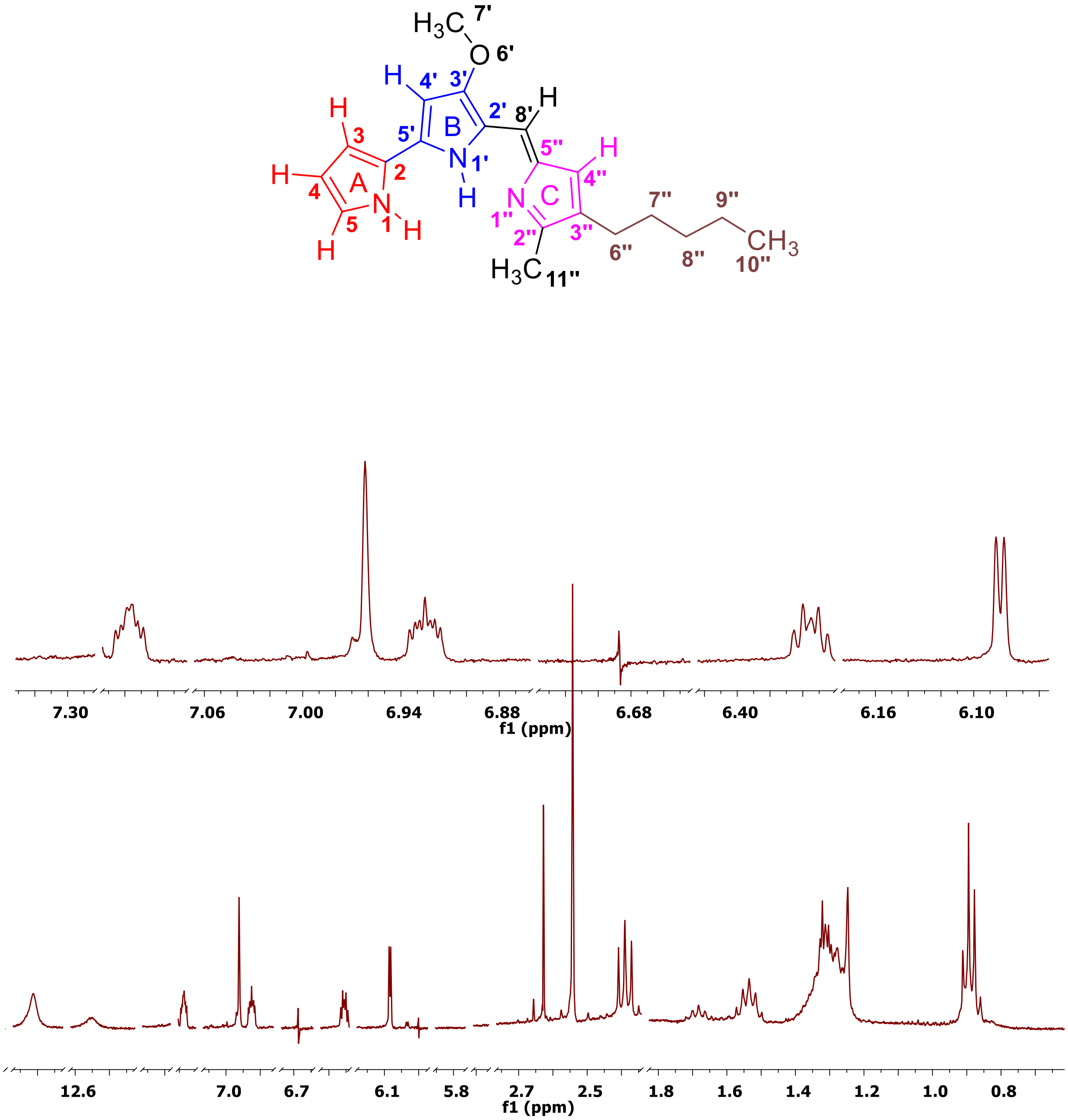

H_3C 7'
O 6'
H 4' 3'
H 3
2' 8' H
5' B
5'' H
H 4 A 2 N 1'
N H 4''
1'' C 7'' 9''
5 N 1 H 2'' 3''
H 6'' 8'' CH_3 10''
H_3C 11''
7.30 7.06 7.00 6.94 6.88 6.68 6.40 6.16 6.10
f1 (ppm)
12.6 7.0 6.7 6.1 5.8 2.7 2.5 1.8 1.6 1.4 1.2 1.0 0.8
f1 (ppm)

E

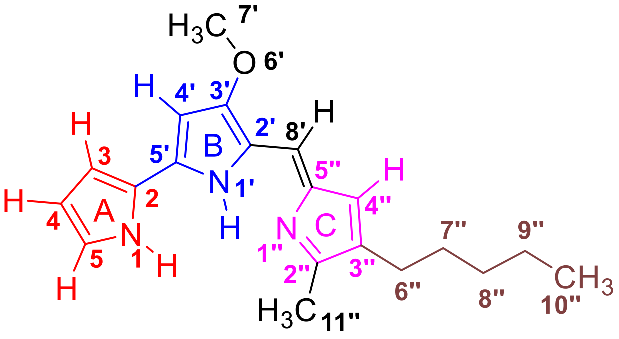
H3C
7'
O 6'
H
4'
3'
2'
8'
H
H
3
5'
B
5''
H
H
4
A
2
N
1'
4''
N
H
7''
9''
1''
C
5
1
H
2''
3''
CH3
H
6''
8''
10''
H3C
11''

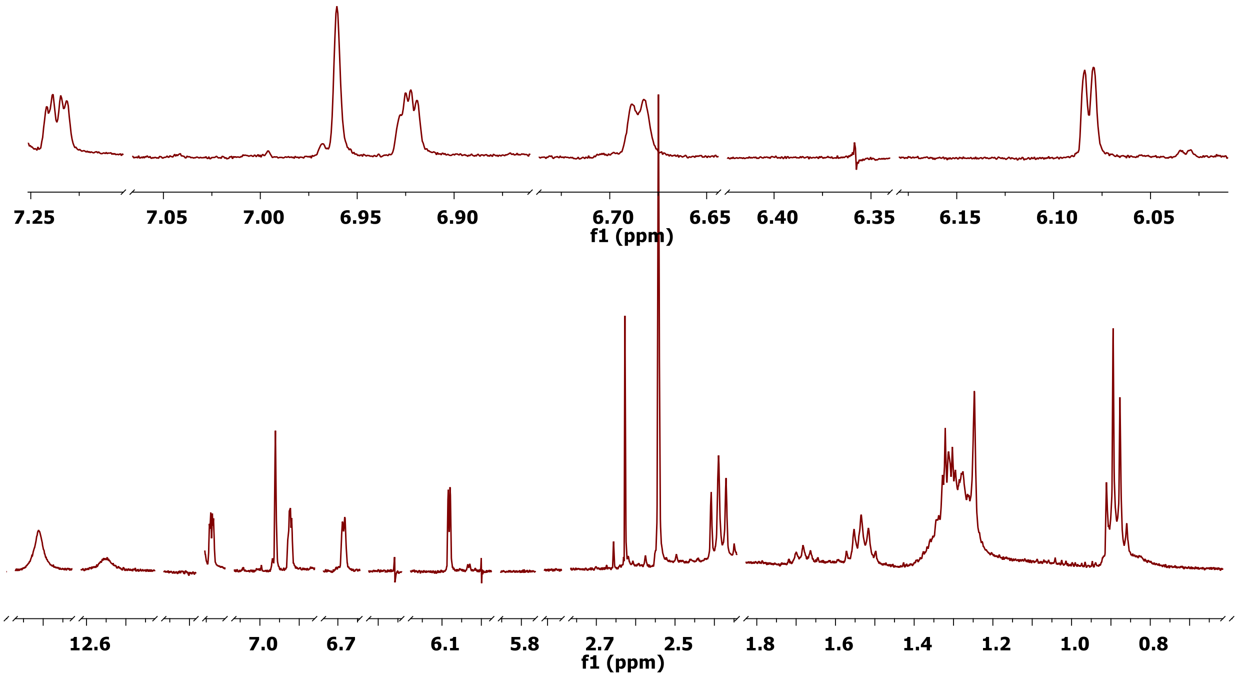
7.25
7.05
7.00
6.95
6.90
6.70
6.65
6.40
6.35
6.15
6.10
6.05
f1 (ppm)
12.6
7.0
6.7
6.1
5.8
2.7
2.5
1.8
1.6
1.4
1.2
1.0
0.8
f1 (ppm)

F

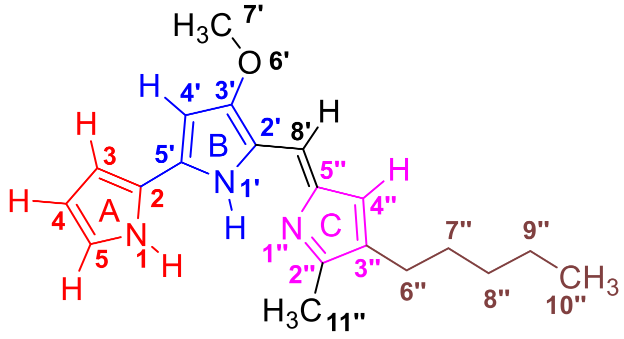

H₃C 7'
O 6'
H 4' 3'
H 3 5' B 2' 8' H
H 4 A 2 N 1' 5'' H
5 N 1 H H N C 4''
1'' 2'' 3'' 7'' 9''
H H₃C 11'' 6'' 8'' CH₃ 10''

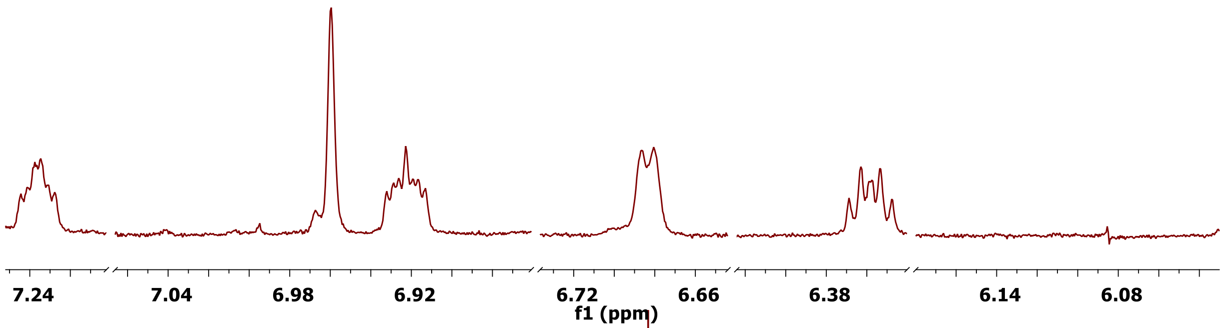

7.24
7.04
6.98
6.92
6.72
6.66
6.38
6.14
6.08
f1 (ppm)

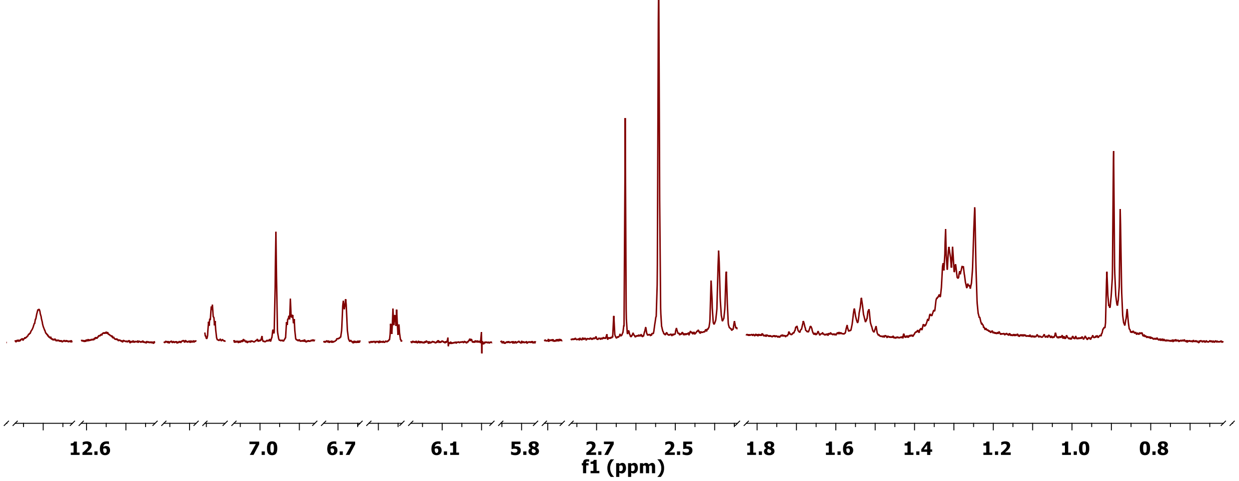

12.6
7.0
6.7
6.1
5.8
2.7
2.5
1.8
1.6
1.4
1.2
1.0
0.8
f1 (ppm)

G

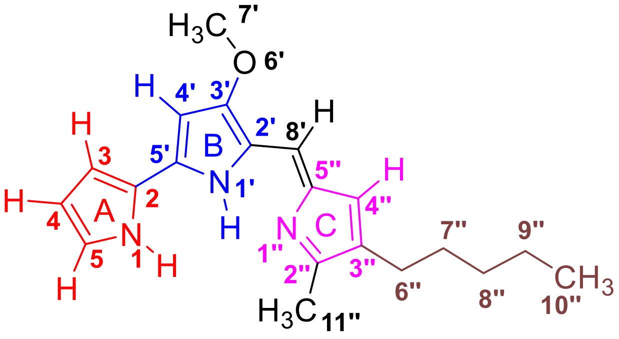

H3C 7'
O 6'
H 4' 3'
2' 8' H
H 3 5' B
5''
H
H 4 A 2 N 1'
H
N C 4''
7'' 9''
5 1 H
1'' 2'' 3''
CH3
H 6'' 8'' 10''
H3C 11''

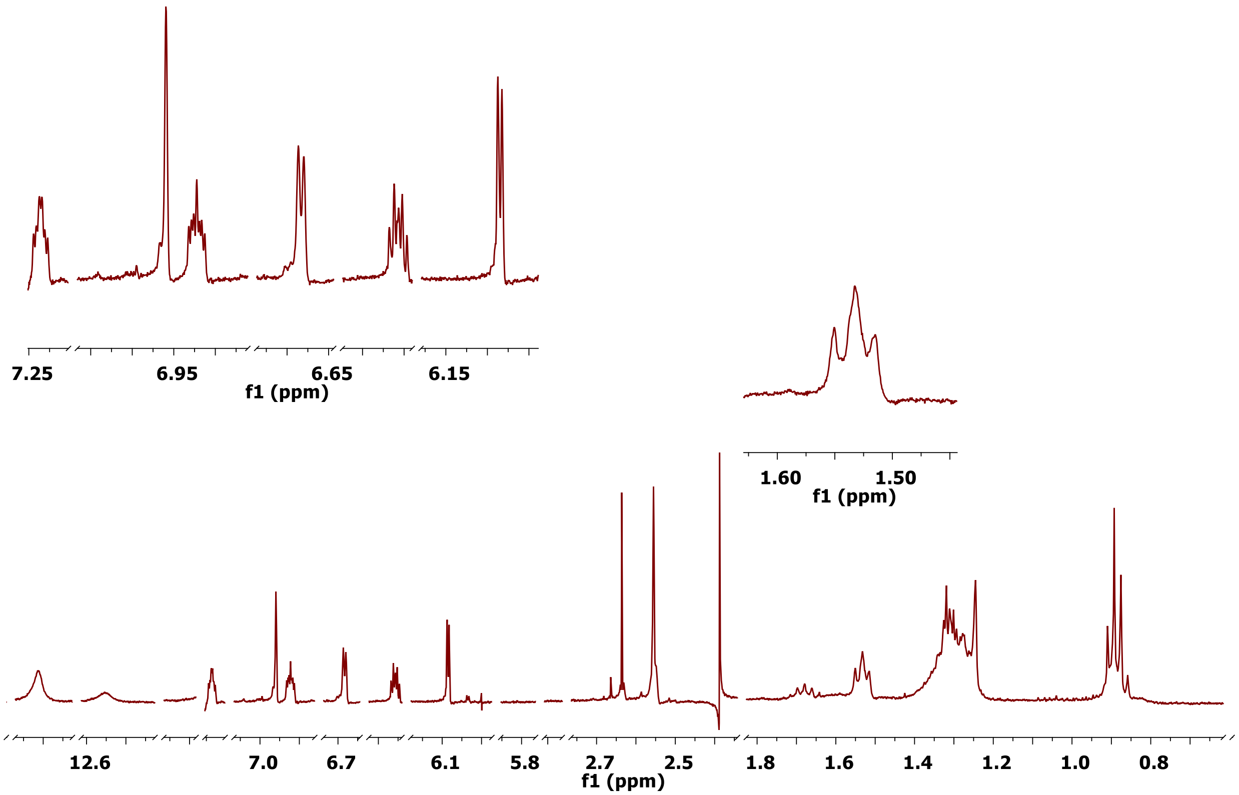

7.25
6.95
6.65
6.15
f1 (ppm)
1.60
1.50
f1 (ppm)
12.6
7.0
6.7
6.1
5.8
2.7
2.5
1.8
1.6
1.4
1.2
1.0
0.8
f1 (ppm)

H

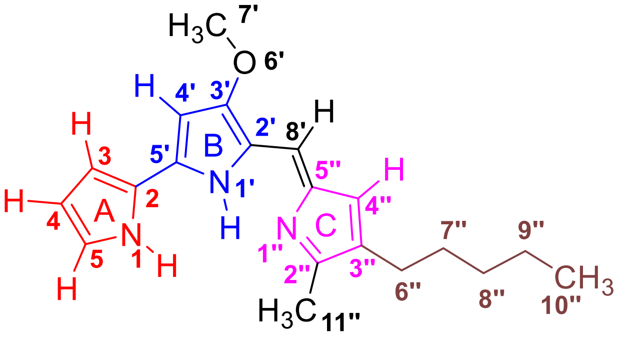

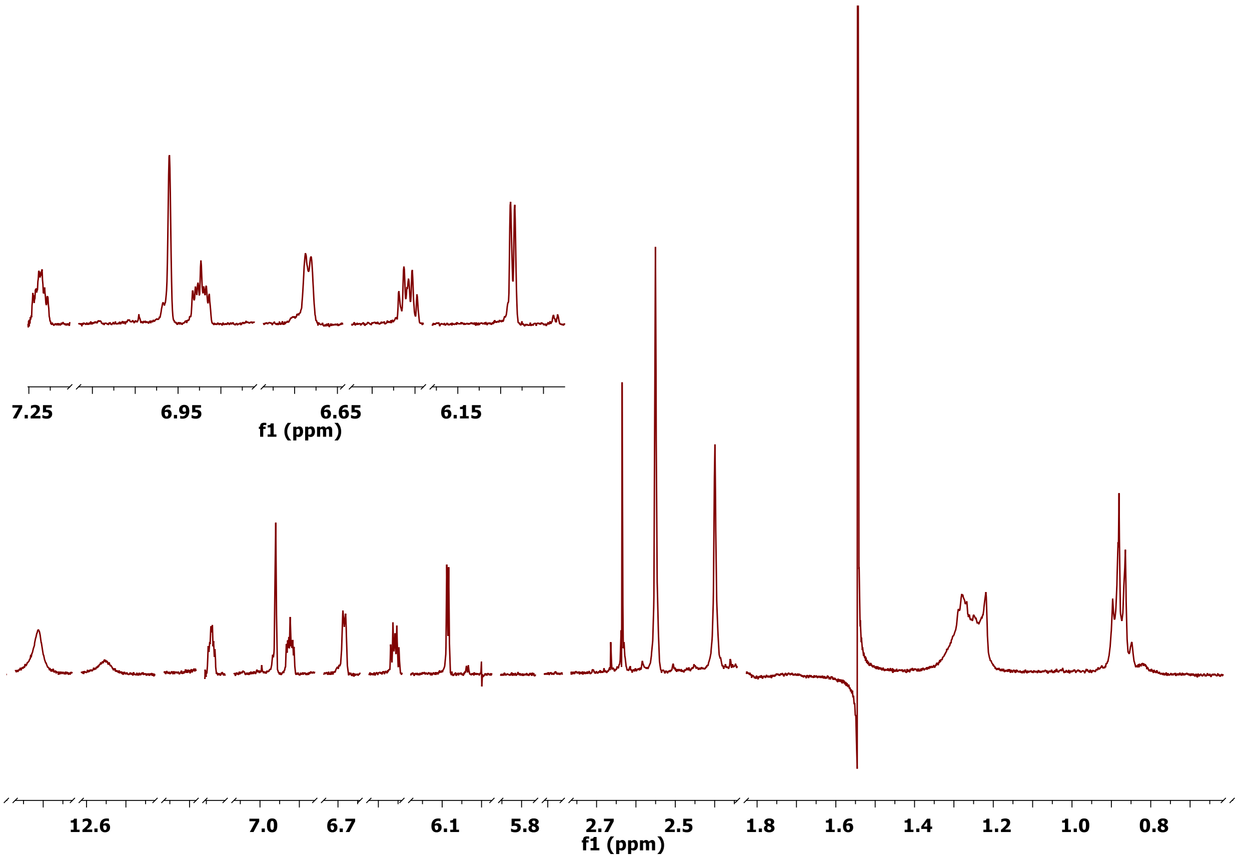

I

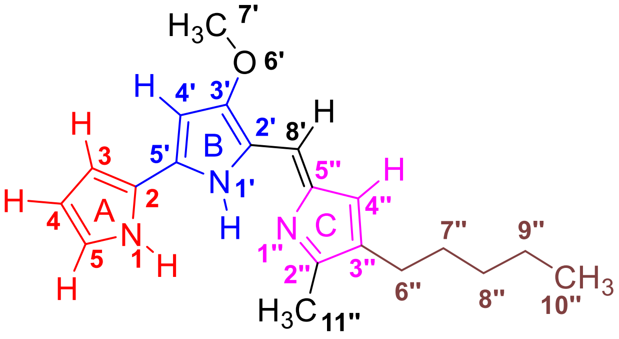
H3C
7'
O
6'
H
4'
3'
2'
8'
H
H
3
5'
B
5''
H
H
4
A
2
N
1'
4''
N
H
C
7''
9''
1
H
1''
2''
3''
5
H
6''
8''
CH3
10''
H3C
11''

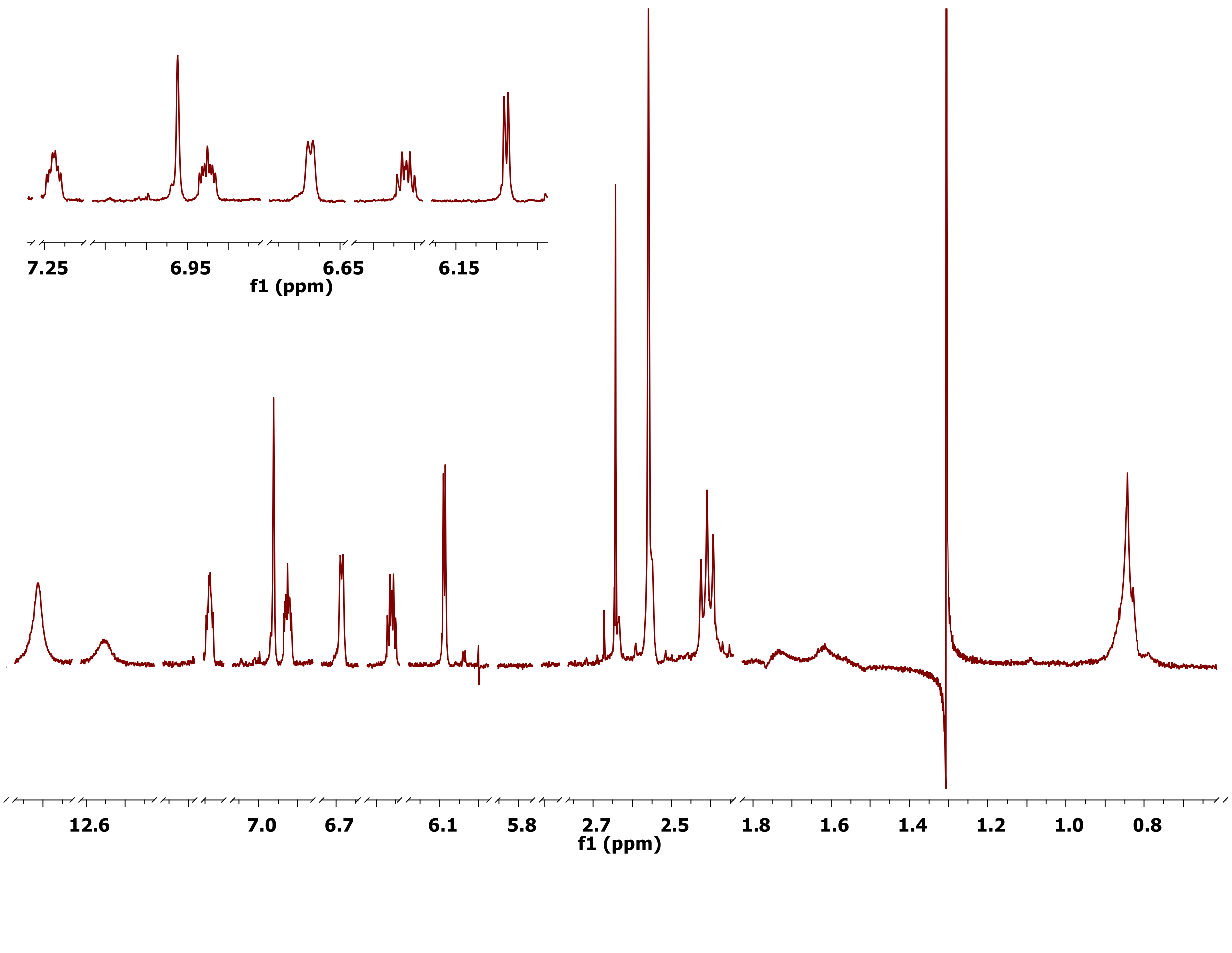
7.25
6.95
6.65
6.15
f1 (ppm)
12.6
7.0
6.7
6.1
5.8
2.7
2.5
1.8
1.6
1.4
1.2
1.0
0.8
f1 (ppm)

J

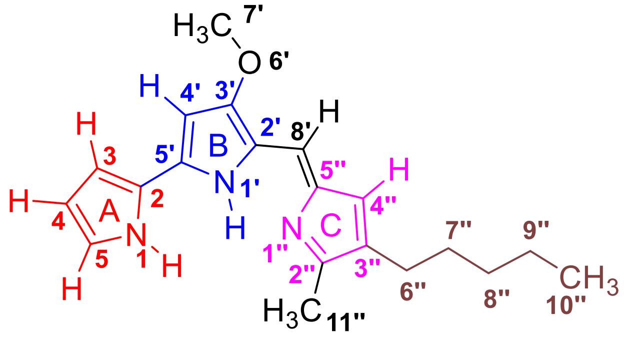
H3C
7'
O
6'
H
4'
3'
2'
8'
H
H
3
5'
B
5''
H
H
4
A
2
N
1'
4''
N
H
C
7''
9''
1''
3''
CH3
5
1
H
2''
H
H3C
11''
6''
8''
10''

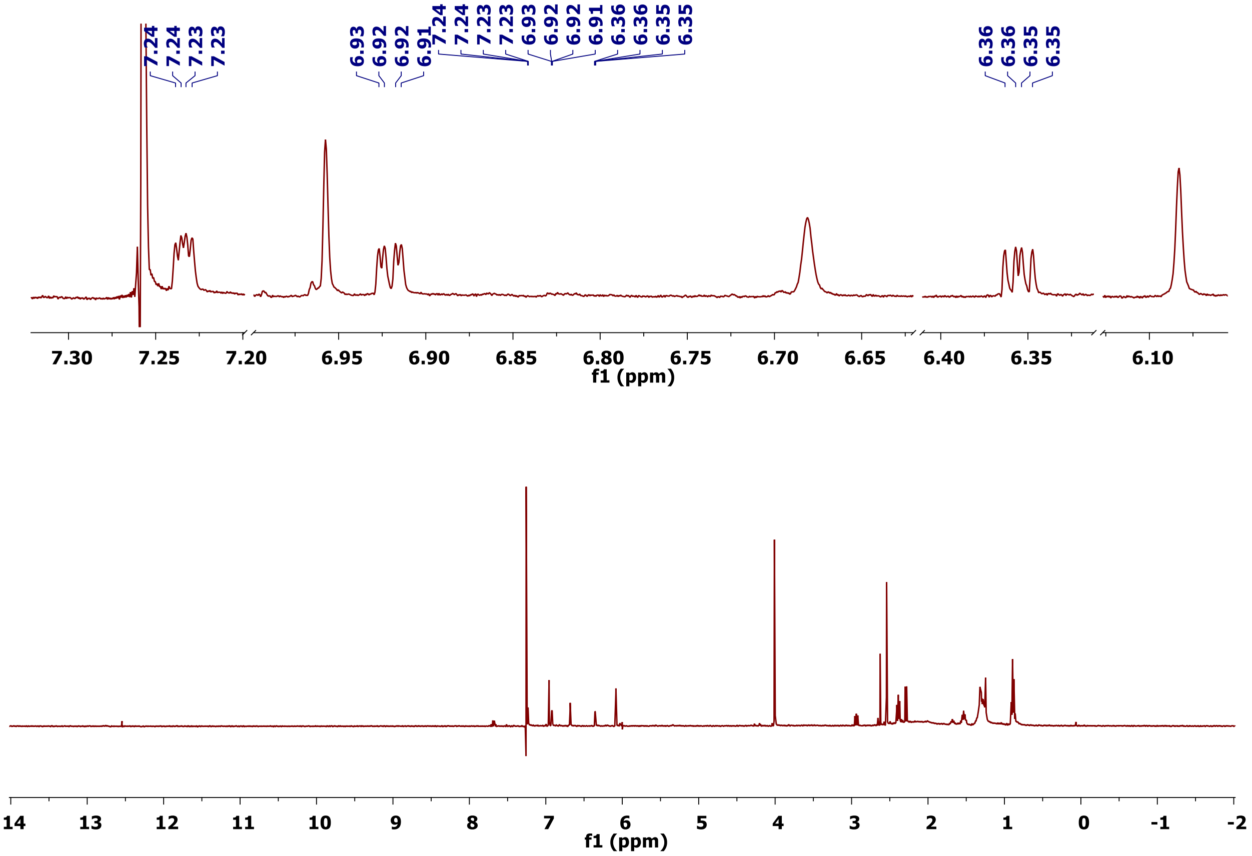
7.24
7.24
7.23
7.23
6.93
6.92
6.92
6.91
7.24
7.24
7.23
7.23
6.93
6.92
6.92
6.91
6.36
6.36
6.35
6.35
6.36
6.36
6.35
6.35
7.30
7.25
7.20
6.95
6.90
6.85
6.80
6.75
6.70
6.65
6.40
6.35
6.10
f1 (ppm)
14
13
12
11
10
9
8
7
6
5
4
3
2
1
0
-1
-2
f1 (ppm)

K

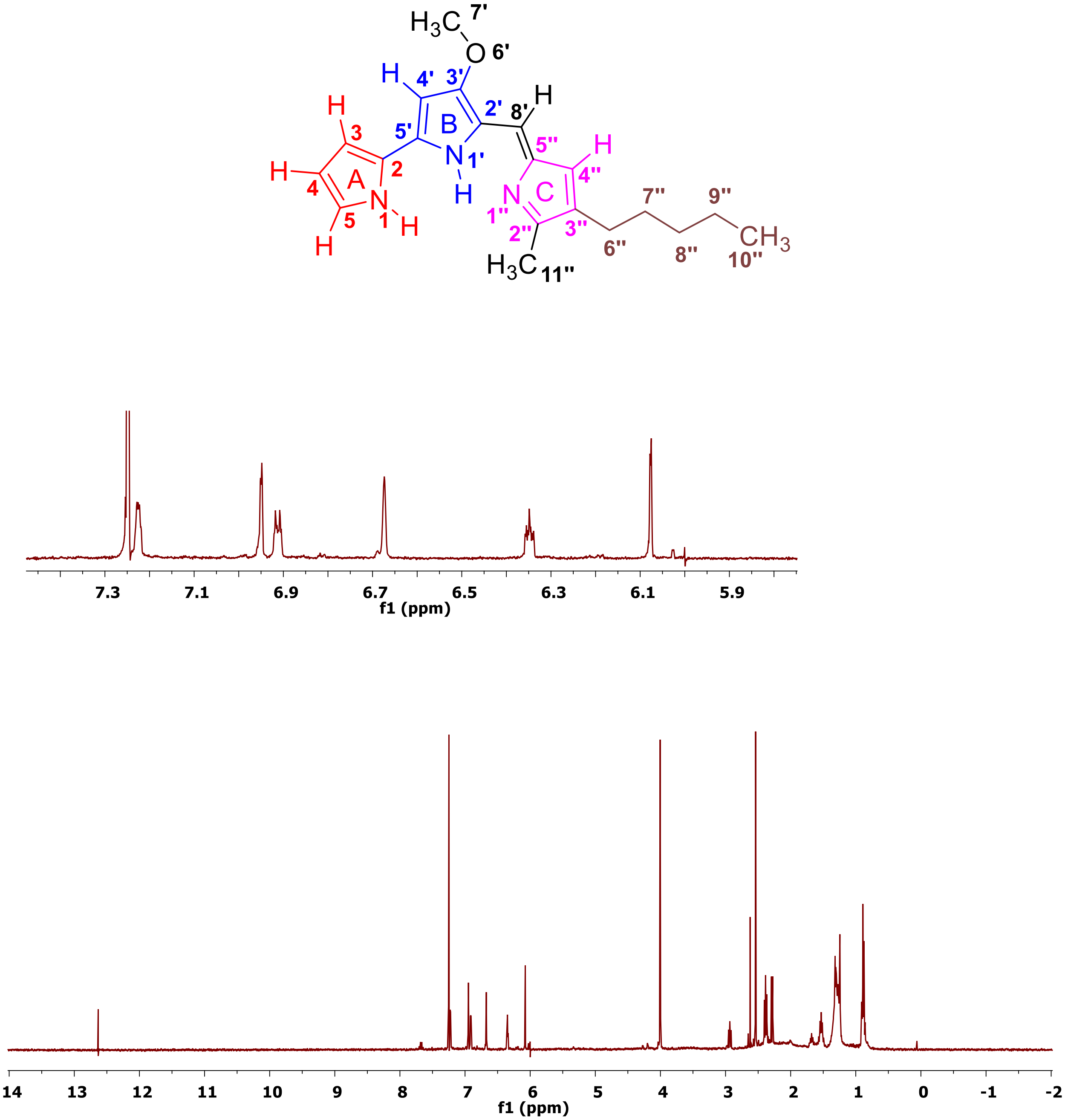

Figure S10. ^{1}H-^{1}H-Homonuclear decoupled NMR spectrum of prodigiosin. Irradiation of: (**A**) C_5-H, (**B**) $C_{4''}$-H, (**C**) C_3-H, (**D**), $C_{8'}$-H, (**E**) C_4-H, (**F**) $C_{4'}$-H, (**G**) $C_{6''}$-H_2, (**H**) $C_{7''}$-H_2 (**I**) $C_{8''}$-H_2 and $C_{9''}$-H_2, (**J**) N_1-H, (**K**) $N_{1'}$-H.
